# Rapid synthesis of glycosylated insulins by flow-based peptide synthesis†

**DOI:** 10.1039/d5sc01670c

**Published:** 2025-04-04

**Authors:** Yuta Maki, Surin K. Mong, Chaitra Chandrashekar, Briony E. Forbes, Mohammed Akhter Hossain, Shintaro Yamaguchi, Colin M. Fadzen, Yasuhiro Kajihara, Bradley L. Pentelute

**Affiliations:** a Department of Chemistry, Graduate School of Science, Osaka University 1-1 Machikaneyama Toyonaka Osaka 560-0043 Japan makiyt11@chem.sci.osaka-u.ac.jp; b Forefront Research Center, Graduate School of Science, Osaka University 1-1 Machikaneyama Toyonaka Osaka 560-0043 Japan; c Department of Chemistry, Massachusetts Institute of Technology Cambridge Massachusetts 02139 USA blp@mit.edu; d The Florey Institute of Neuroscience and Mental Health, The University of Melbourne Victoria 3010 Australia; e Discipline of Medical Biochemistry, College of Medicine and Public Health, Flinders University Bedford Park South Australia 5042 Australia; f The Koch Institute for Integrative Cancer Research, Massachusetts Institute of Technology Cambridge Massachusetts 02142 USA; g Center for Environmental Health Sciences, Massachusetts Institute of Technology Cambridge Massachusetts 02139 USA; h Broad Institute of MIT and Harvard Cambridge Massachusetts 02142 USA

## Abstract

Insulin is a key life-saving drug for patients with diabetes and is used clinically worldwide. To address the physicochemical challenges of insulin, such as low solubility and aggregation, glycosylated insulins have been chemically synthesized, exhibiting improved stability due to the hydration effect of glycans. In this work, we demonstrated the rapid synthesis of glycosylated insulins (glycoinsulins) using flow-based solid-phase peptide synthesis (SPPS). The insulin A-chain and glycosylated B-chain were synthesized by flow-based SPPS, with each elongation cycle completed in just 3 minutes. Through our investigations, the glycosylation step was successfully performed within 10 minutes under optimized flow-based conditions. Additionally, we examined the incorporation of dipeptide units (isoacyl dipeptide and pseudoproline) under flow conditions and demonstrated efficient peptide elongation by combining flow-based SPPS with these dipeptide units. The synthesized A- and B-chains were subsequently used for the stepwise formation of disulfide bond linkages. The resulting glycoinsulins exhibited comparable binding affinities to insulin receptors. These findings highlight a novel flow-based approach for the rapid synthesis of glycosylated peptide and protein drugs.

## Abstract 2

Accelerated production of insulin and glycosylated insulins by a rapid flow-based approach.

## Graphical abstract

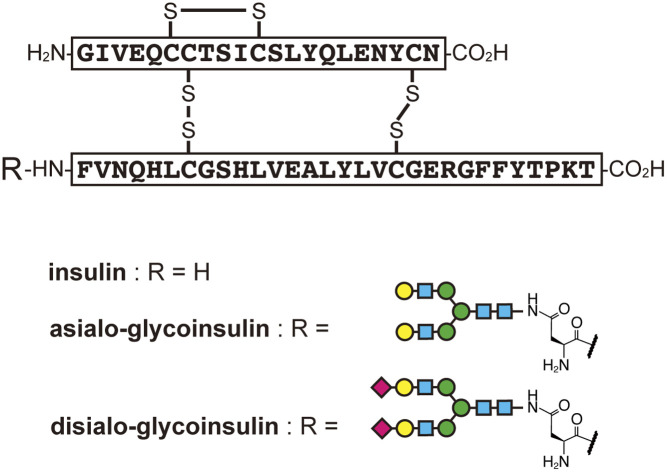

## Introduction

Glycosylation of proteins is a major post-translational modification that alters the structure, stability, and biological activity of proteins.^1^ In the case of commercial biologics, glycosylation is critical for inducing the inherent activity of proteins. For instance, immunoglobulin G (IgG) antibodies and erythropoietin are modified with asparagine-linked (*N*-)glycans, and the structures of these *N*-glycans are essential for antibody-dependent cellular cytotoxicity (ADCC) and hematopoietic activity.^2^

In addition, glycan engineering of proteins and peptides has been extensively studied. Artificially increasing the number of *N*-glycans has been shown to prolong the blood circulation lifetime of erythropoietin glycoprotein, leading to the development of the glycoprotein drug darbepoietin, which is widely used for treating anemia.^3^ Furthermore, a recent study reported that the metabolic stability of the peptide somatostatin (somatotropin release-inhibiting factor, SRIF) was enhanced by the artificial introduction of a human complex-type glycan.^4^

**Fig. 1:**
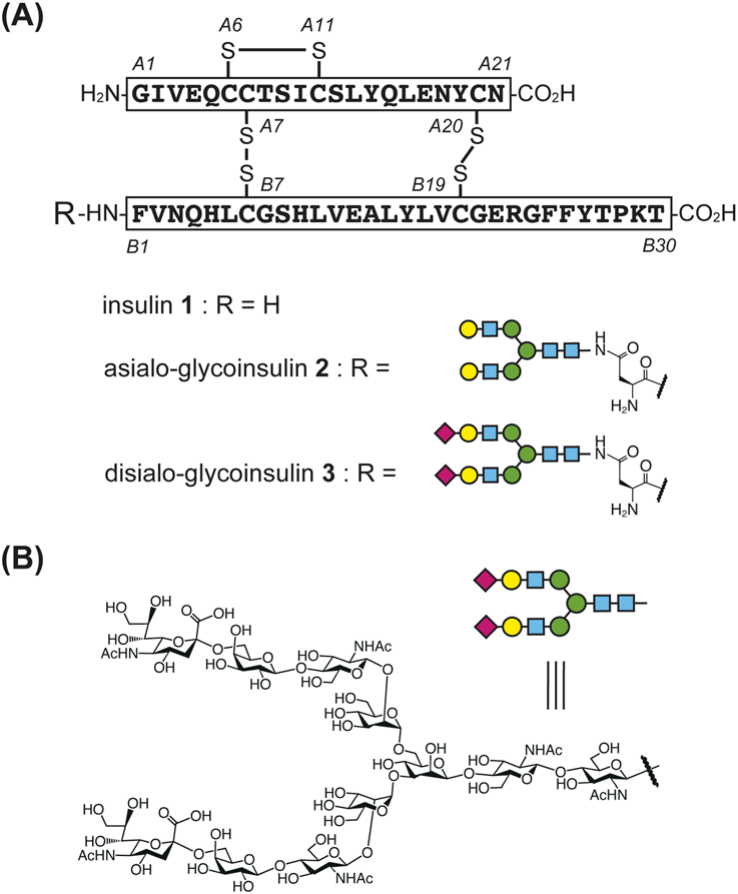
(A) Structures of native human insulin and artificially glycosylated insulin derivatives (glycoinsulins). (B) Structure of human asparagine-linked (*N*-)glycan.

Insulin and its analogs have been widely targeted for synthesis by many research groups due to their importance as peptide hormone drugs and the uniqueness of their chemical structural features.^5^ Human insulin 1 is a key life-saving drug for diabetic patients. It is produced by beta cells in the pancreas, and its primary structure consists of two peptide chains: a 21-amino acid A-chain and a 30-amino acid B-chain (Fig. 1A).^6^ The A-chain contains an internal disulfide bond (A6–A11) and is linked to the B-chain *via* two interchain disulfide bonds (A7–B7 and A20–B19). To efficiently achieve the three disulfide bonds, several strategies have been developed, including random combination among six cysteine thiols,^5*c*^ single-chain proinsulin-type folding followed by peptidase digestion,^7^ and stepwise disulfide bond formation by orthogonal protections.^8^

The artificial introduction of glycans into insulin is a promising approach to improving its activity and chemical properties.^9^ Several research groups have reported the synthesis of glycosylated insulins, referred to as glycoinsulins.^10^ Notably, Hossain *et al.* introduced a homogeneous human-type *N*-glycan to the N-terminus of the B-chain *via* nonnative thioether linkage, as this position was considered to have minimal impact on the binding properties with the insulin receptor.^10*e*^ Artificial glycosylation significantly improved insulin's stability and inhibited its aggregation without causing a notable decrease in receptor binding affinity. Since fibril formation is undesirable and reduces the shelf life of insulin drugs, glycosylation represents a potentially valuable strategy for developing improved protein-based therapeutics.

Solid-phase peptide synthesis (SPPS) is a powerful method for preparing desired peptide sequences.^11^ This approach also enables the site-specific incorporation of noncanonical amino acids, including glycan-linked amino acids.^12^ In the case of 9-fluorenylmethyloxycarbonyl (Fmoc)-SPPS, each peptide elongation step requires several reactions including amino acid coupling, Fmoc group deprotection, and resin washing steps.^13^ Each elongation cycle, including the repetitive washing steps, requires *ca.* 5–60 minutes depending on conditions and equipment, as SPPS methodologies have been improved through advancements such as automated synthesizers and microwave-assisted synthesis.^14^

In glycopeptide synthesis, glycosyl-amino acids, whether isolated or synthetically prepared, can serve as building blocks in SPPS.^15^ Although this method enables the site-specific introduction of glycans,^16^ the overall process requires significant synthetic effort and time, particularly for longer peptides with “difficult sequences”, aggregation-prone regions, or low solubility. Thus, efficient methods to synthesize glycopeptides are required for applied researches, such as improving peptide-based drugs and synthesizing glycosylated protein derivatives.

In this work, we investigated flow-based coupling of glycosyl-asparagine and dipeptide units (isoacyl dipeptide and pseudoprolione) to rapidly synthesize glycosylated insulin derivatives (glycoinsulins). Under manual flow conditions,^17^ peptides were synthesized rapidly, as each elongation step was completed within 3 minutes, including amino acid coupling (<40 seconds), DMF washing (60 seconds), Fmoc deprotection (20 seconds), and another DMF washing step (60 seconds). Compared to previous methods for insulin synthesis, this flow chemistry accelerated the production of insulin A- and B-chains.

We also optimized several flow-based conditions for introducing glycosyl-asparagine, isolated from a natural source,^15*a*^ at the N-terminus of the B-chain. We hypothesized that glycosyl-asparagine could be rapidly incorporated into the B-chain *via* a simple amide bond formation in the reactor of the flow system. Additionally, we investigated the introduction and utility of dipeptide units (isoacyl dipeptide and pseudoproline) within the flow-based SPPS process.

While carbohydrate modification of insulin has been reported by several groups,^9,10^ we successfully demonstrated, for the first time, the synthesis of glycoinsulins containing two human-type glycans linked through the asparagine side chain.

## Results and discussion

**Fig. 2:**
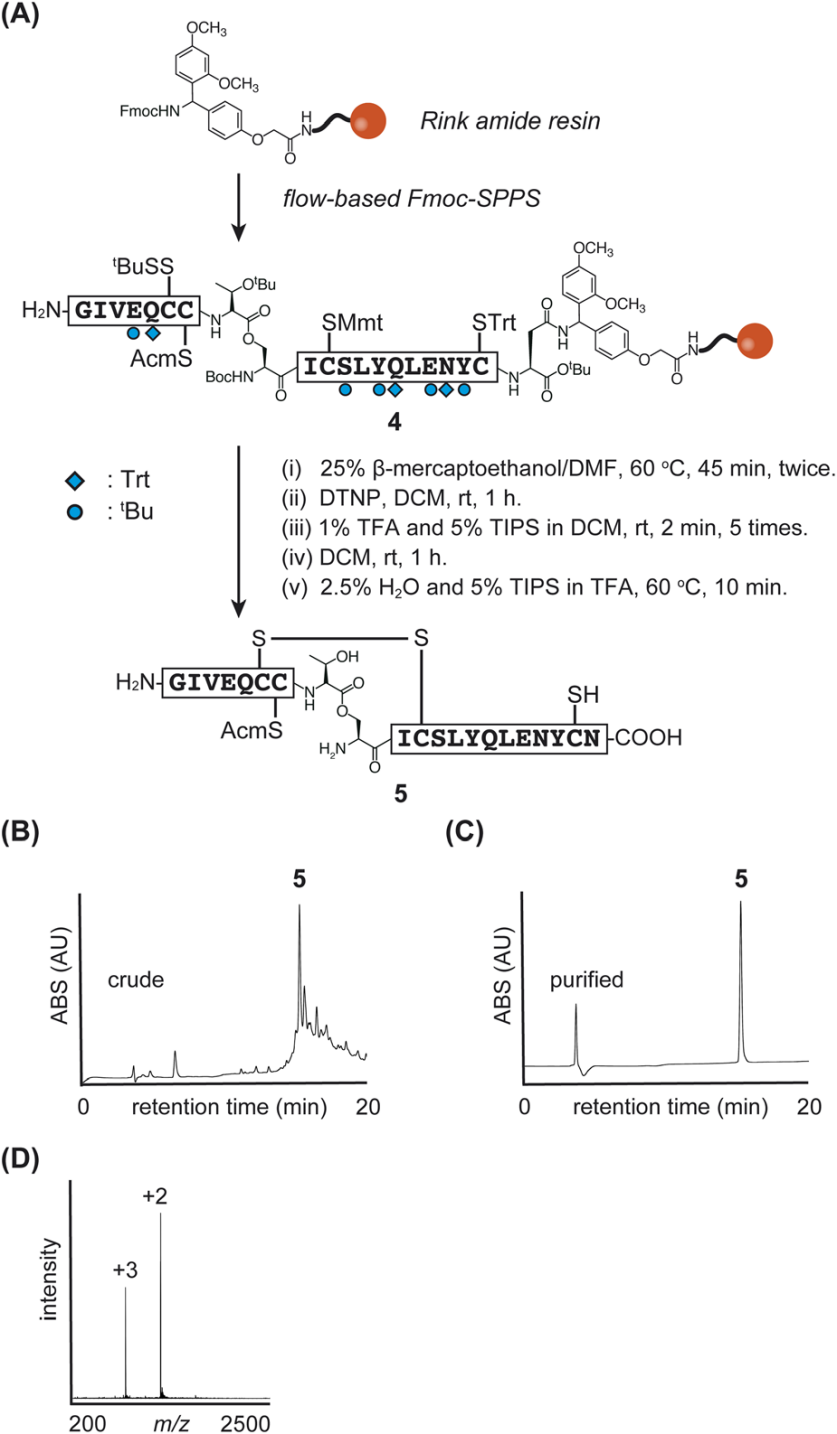
(A) Synthetic scheme of insulin A-chain 5. (B) and (C) HPLC monitoring. (D) Direct infusion data of purified peptide 5.

The synthetic strategy for the insulin A-chain is shown in Fig. 2. The A-chain is known to have poor solubility.^5*c*,18^ To address this issue, we incorporated an isoacyl dipeptide unit at the Thr^A8^-Ser^A9^ position (superscripts indicate positions in the A-chain), following a previous report,^8*b*^ as the isoacyl dipeptide improves both peptide solubility and synthetic efficiency.^19^

For the protection of cysteine residues, we employed S^*t*^Bu, acetamidomethyl (Acm), *p*-methoxytrityl (Mmt), and Trt at positions-6, -7, -11, and -20, respectively. These protecting groups were chosen based on their reported effectiveness in enhancing the solubility and facilitating the purification of the A-chain.^8*b*^ Additionally, we considered these cysteine protecting groups to be suitable for glycoinsulin synthesis, as they avoid the need for highly acidic and oxidative conditions after glycan introduction.

We synthesized the protected A-chain 4 and investigated the flow-coupling of the isoacyl dipeptide. First, the peptide was elongated using the manual flow-based SPPS, with a 3 minute cycle each time, except for the dipeptide coupling step. The reactions were conducted at 60 °C using hexafluorophosphate azabenzotriazole tetramethyl uronium (HATU) as the coupling reagent. Next, standard flow conditions employing HATU or 2-(1*H*-benzotriazole-1-yl)-1,1,3,3-tetramethyluronium hexafluorophosphonate (HBTU) along with *N*,*N*-diisopropylethylamine (DIEA) were tested for dipeptide coupling. However, unidentified byproducts were observed during LC/MS monitoring after the coupling reaction. In contrast, efficient dipeptide coupling was achieved under standard batch conditions at room temperature using various reagents, including 1-hydroxybenzotriazole (HOBt)/*N*,*N*′-diisopropylcarbodiimide (DIC), HBTU/DIEA, HATU/DIEA, and (benzotriazol-1-yloxy)tripyrrolidinophosphonium hexafluorophosphate (PyBOP)/DIEA. These results suggested that the heated flow conditions might not be suitable for this specific coupling reaction.

Since the standard flow coupling requires a fixed amount of Fmoc-amino acid (1 mmol),^17^ we concluded that the costly dipeptide should be coupled outside of the flow system to allow flexible adjustment of its quantities depending on the synthetic scale. Ultimately, the dipeptide unit was incorporated by DIC/HOBt at room temperature for 1 hour under non-flow conditions outside of the flow reactor, while other amino acids were coupled using flow-based methods. The synthetic efficiency was confirmed by preliminary test cleavage (Fig. S1†).

The peptide 4 was elongated within 2 hours, representing a significant reduction in time compared to traditional manual SPPS methods. Our synthesis using the rapid manual flow-SPPS enabled the elongation to be completed in just 2 hours. This accelerated process also allowed us to repeat the synthesis efficiently to obtain sufficient quantities of the desired material.

Next, we investigated the manipulation of cysteine protecting groups in the A-chain of peptide 4 on the resin, following a previously reported method with slight modifications.^8*b*^ The S^*t*^Bu group of Cys^A6^ was removed using 25% 2-mercaptoethanol in DMF at 60 °C, and the resulting Cys^A6^-thiol was activated with 5-nitropyridyl sulfide. The Mmt group of Cys^A11^ was selectively removed using 1% TFA and 5% TIPS in DCM to form the internal disulfide bridge (Cys^A6^-Cys^A11^). Finally, treatment with a TFA cocktail yielded the desired A-chain peptide 5 with Cys^A20^-SH (yield 4%, calculated from resin), as shown in Fig. 2.

**Fig. 3:**
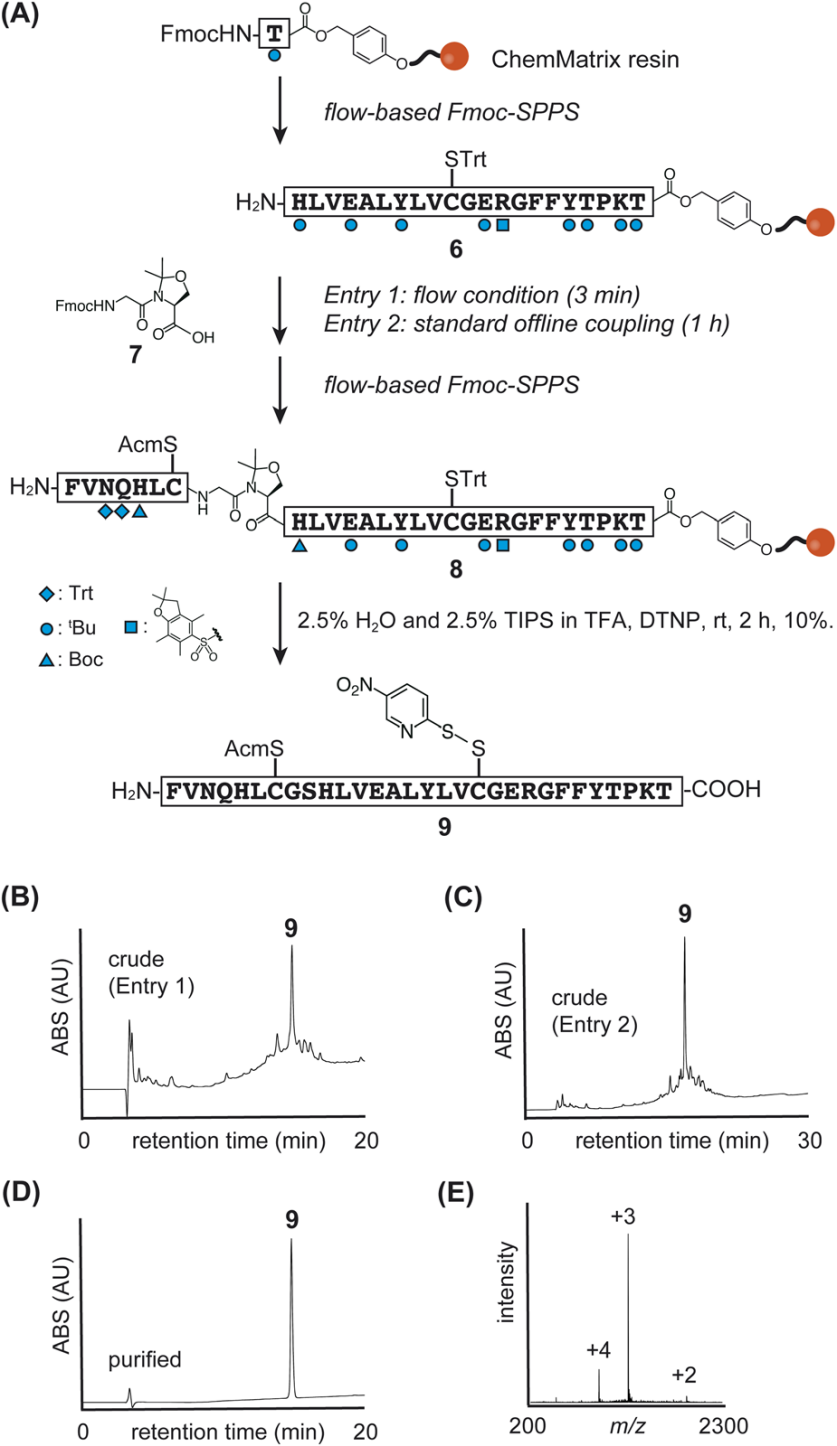
(A) Synthetic scheme of insulin B-chain 9. (B)–(D) HPLC monitoring. (E) Direct infusion data of purified peptide 9.

We then focused on optimizing the synthesis of a non-glycosylated insulin B-chain using the flow-SPPS and the pseudoproline dipeptide 7 (Fig. 3A). Initial investigations revealed challenges with peptide elongation in the N-terminal region (Fig. S4†). To address this challenge, we introduced the pseudoproline dipeptide 7 at the Gly^B8^-Ser^B9^ position. However, amino acid deletions persisted, specifically at Leu^B11^, Val^B12^, Glu^B13^, and Ala^B14^, potentially due to the aggregation properties of the peptide on the resin (Fig. S4†). To mitigate this issue, the coupling time was extended and Fmoc group deprotection was repeated to ensure reaction efficiency for Leu^B11^, Val^B12^, Glu^B13^, and Ala^B14^ (ESI†).

Regarding the coupling of pseudoproline dipeptide 7, we tested two conditions: standard flow coupling (3 minute cycle, Fig. 3A, entry 1) and standard offline coupling (1 hour, Fig. 3A, entry 2). We found that both conditions facilitated the efficient synthesis of the insulin B-chain (Fig. 3, S2, and S3†).

After peptide elongation, 4-nitropyridyl sulfide was introduced to the thiol group of Cys^B19^ using 2,2′-dithiobis(5-nitropyridine) (DTNP) in the TFA cocktail during the global deprotection step, yielding the desired B-chain 9 (yield 10%, calculated based on the loading of Fmoc-Thr^B30^). Following these optimizations, we observed an appropriate crude LC chromatogram, as shown in Fig. 3B and C.

Flow-SPPS enabled the rapid elongation of the B-chain peptide. When the dipeptide unit was incorporated under standard flow conditions, the peptide elongation time from Thr^B30^-HMPA-resin was reduced to 1.7 hours.

**Fig. 4:**
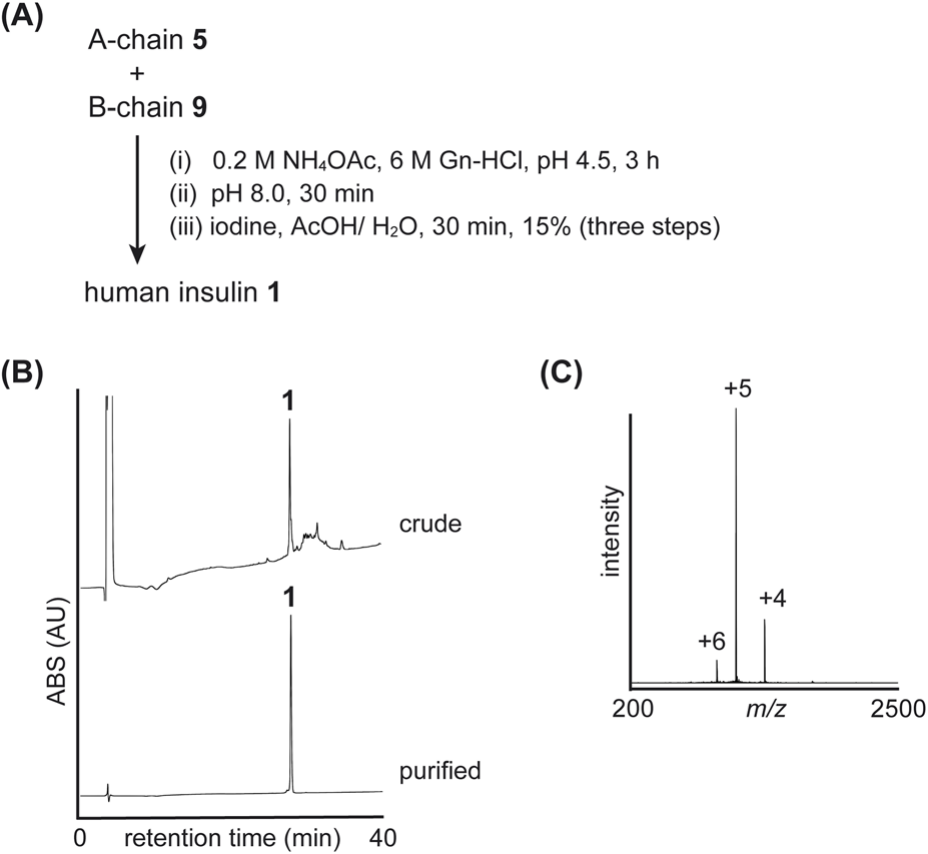
Folding of native insulin: (A) reaction scheme. (B) HPLC monitoring. (C) Direct infusion analysis of purified insulin 1.

Using insulin A-chain 5 and B-chain 9, we examined the folding reaction (Fig. 4 and S11†). First, synthetic A-chain 5 and B-chain 9 were dissolved in NH_4_OAc buffer (pH 4.5) to form an interchain disulfide bond (peptide S5 with Cys^A20^-Cys^B19^). Next, the pH of the mixture was raised to 8.0 to convert the ester bond of Thr^A8^-Ser^A9^ into the native amide bond. This conversion was confirmed by comparing the retention times of the peptides (S5 and S6), where the native amide bond form (peptide S6) eluted later than the isoacyl bond form (peptide S5), as previously reported.^8*b*^ Finally, the Acm groups of Cys^A7^ and Cys^B7^ were removed, and an interchain disulfide bond was simultaneously formed through the addition of iodine. These reactions were performed in a one-pot manner to yield the desired human insulin 1 (yield 15%, three steps, calculated from B-chain 9).

To confirm the regioselective formation of disulfide bonds, we performed peptidase digestion using endoproteinase Glu-C, which specifically cleaves amide bonds at the carboxy side of Glu residues (Fig. S18†). Since the A-chain contains consecutive Cys residues at position-6 and -7, we prepared peptide isomers as standards (Fig. S15–S17†) and compared their retention times with those of the digested fragments from synthetic insulin 1. The results confirmed that our synthetic insulin contained the desired disulfide bond pairs.

**Fig. 5:**
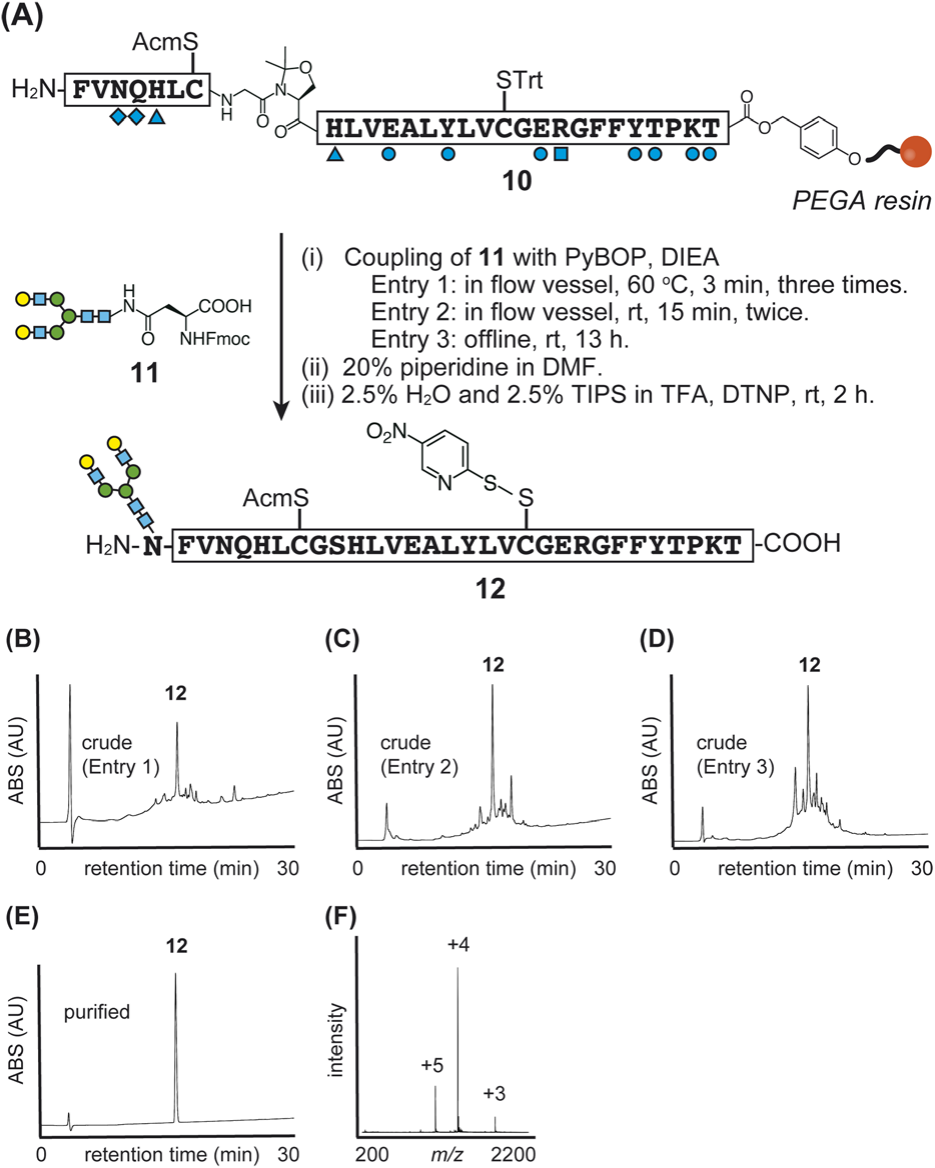
(A) Synthetic scheme of glycosylated B-chain 12. (B)–(E) HPLC data for crude (entry 1, 2, and 3) and purified 12. (F) Direct infusion analysis of purified glycosylated B-chain 12.

Since we successfully established the synthesis of native human insulin using flow-SPPS, we next examined the synthesis of glycosylated insulin (glycoinsulin), as shown in Fig. 5. In this case, PEGA resin was employed to enable efficient coupling of glycosyl-asparagine,^15*a*^ and peptide elongation was carried out in the same manner as for the non-glycosylated B-chain 9.

After elongating peptide 10 on resin, we examined several conditions for coupling Fmoc-Asn(asialooligosaccharide)-OH 11, which was isolated from egg yolk.^15*a*^ Initially, we tested standard flow-conditions; however, the glycosyl-asparagine was not soluble in DMF at a concentration of 0.4 M due to its hydrophilicity and low solubility in organic solvents.^20^ To address this issue, the concentration of glycosyl-asparagine was reduced (50–200 mM), and coupling reactions were attempted using HBTU or HBTU/HOBt in combination with DIEA. Unfortunately, these reactions were not successful and resulted in multiple peaks (Fig. S8†).

Next, we employed PyBOP as the coupling reagent for glycosyl-asparagine (33 mM) and conducted the reaction in a flow reactor at 60 °C (Fig. 5A, entry 1) and at room temperature (Fig. 5A, entry 2). In both cases, a mixture of the activated glycosyl-asparagine was delivered to the reactor, and the flow was stopped only during the reactions. Since single coupling reactions did not reach completion, repeated couplings were performed: for entry 1, three cycles of 3 minutes each; for entry 2, two cycles of 15 minutes each. Subsequently, global deprotection and protection of Cys^B20^-SH were carried out to yield the desired glycosylated B-chain 12 with yields of 11% and 8%, as shown in Fig. 5B and C.

Additionally, we examined the coupling of glycosyl-asparagine 11 under typical offline conditions using PyBOP in NMP/DMF (entry 3: room temperature, 13 hours, without a flow vessel). However, this method proved to be less reproducible and less efficient compared to previously reported glycopeptide syntheses, potentially due to the aggregative properties of the peptide. Nevertheless, offline coupling remains a useful approach for conserving glycosyl asparagine when its availability is limited.

Although the optimized flow conditions (entries 1 and 2) required a larger amount of glycosyl-asparagine 9, the synthetic time was significantly reduced. For entry 1, the glycosylated peptide was constructed within 1.9 hours from the Thr-HMPA-PEGA resin, whereas the typical offline coupling condition (entry 3) required approximately 16 hours. Furthermore, based on HPLC and NMR analysis (Fig. S9†), no epimerization was observed at the glycosylated asparagine.

**Fig. 6:**
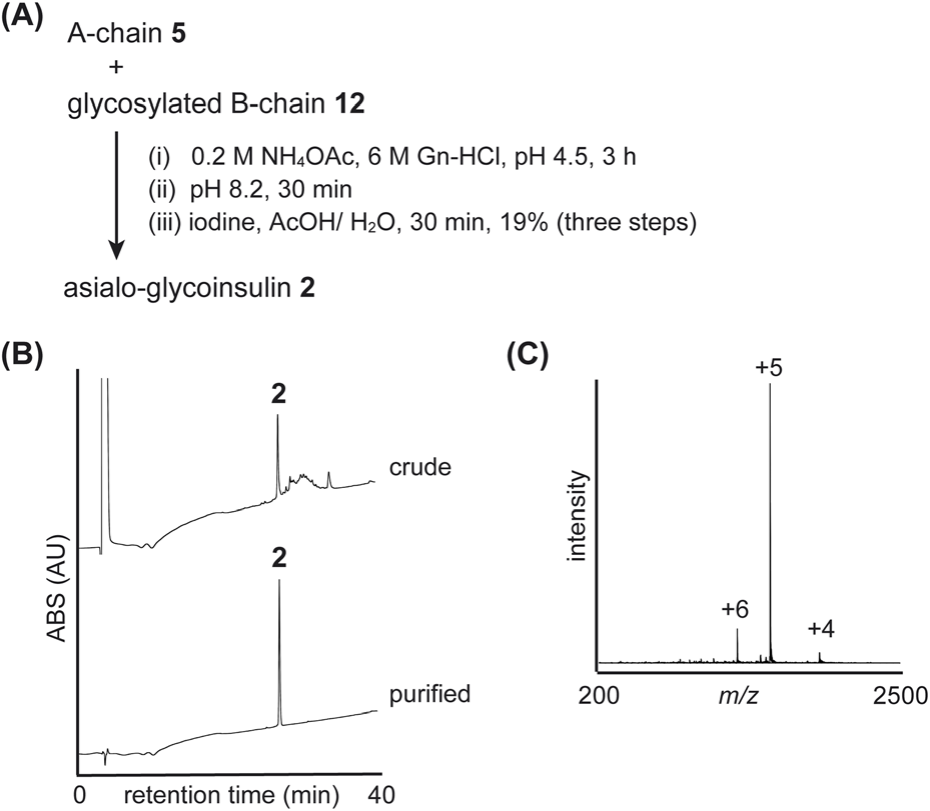
Folding of asialo-glycoinsulin 2. (A) Synthetic scheme. (B) HPLC monitoring. (C) Direct infusion analysis of purified asialo-glycoinsulin 2.

The folding of the asialo-glycoinsulin was then examined (Fig. 6 and S12†). Similar to native insulin, we carried out interchain disulfide bond formation, conversion of the isoacyl bond, and oxidative removal of Acm group to produce the asialo-glycoinsulin 2, as shown in Fig. 6. Based on our observation, the bulky glycan did not sterically hinder the reactions, and the desired asialo-glycoinsulin was obtained through RP-HPLC purification (yield 19%, calculated from the glycosylated B-chain 12).

Next, we examined the synthesis of disialylated glycoinsulin (disialo-glycoinsulin 3), as sialic acid is commonly observed at the non-reducing terminal of *N*-glycans and is known to prolong the half-life of proteins in blood circulation.

**Fig. 7:**
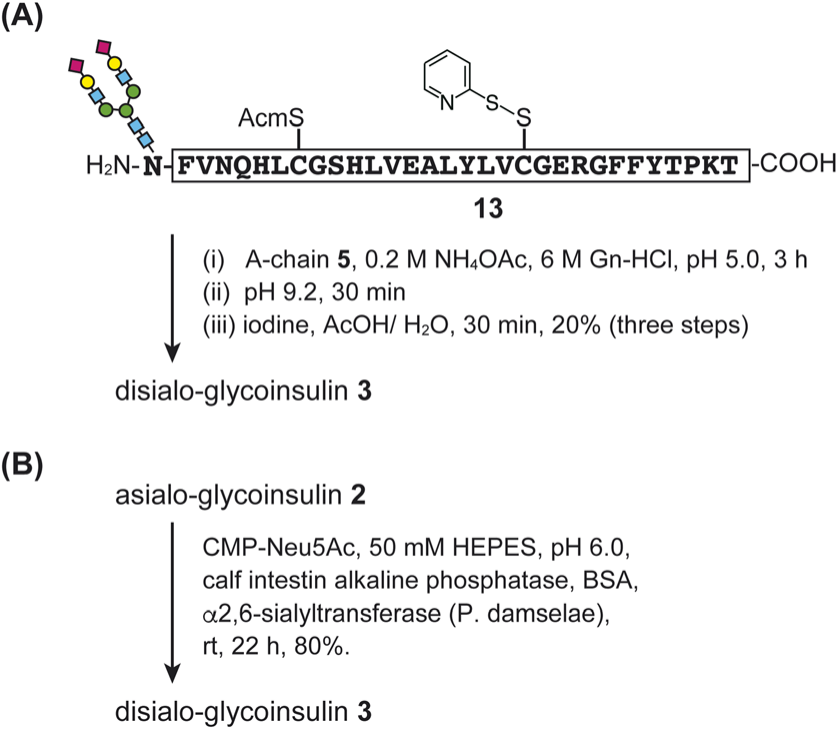
Synthesis of disialo-glycoinsulin 3. (A) Stepwise disulfide bond formation. (B) Enzymatic sialylation.

For the disialo-glycoinsulin 3, we examined two approaches: optimized flow-coupling of sialylated asparagine and enzymatic sialylation (Fig. 7).

First, we investigated the synthesis of disialylated B-chain 13 using flow-based conditions (Fig. S10†). As a building block, we prepared Fmoc-Asn(disialooligosaccharide)-OH S3 with two phenacyl (Pac) esters to protect the carboxylic acids of sialic acid during SPPS and global deprotection. The disialylated B-chain was rapidly prepared in a manner similar to asialo-glycosylated B-chain 12, and the glycosyl-asparagine S3 was incorporated into the B-chain under stopped-flow conditions (15 minutes, repeated three times). Details of the synthesis are provided in the ESI.† Subsequently, folding of disialo-glycoinsulin 3 was performed *via* stepwise disulfide bond formation (Fig. 7A).

Next, we examined enzymatic sialylation. Asialo-glycoinsulin 2 was incubated with bacterial α2,6-sialyltransferase (*P. damselae*) in the presence of CMP-Neu5Ac. The reaction proceeded smoothly in 50 mM HEPES buffer (pH 6.0) containing alkaline phosphatase and bovine serum albumin (BSA). The desired disialo-glycoinsulin 3 was obtained by RP-HPLC purification (yield 80%), as shown in Fig. 7B. Based on our LC and MS analyses, no disulfide bond shuffling was observed during the reaction.

**Fig. 8:**
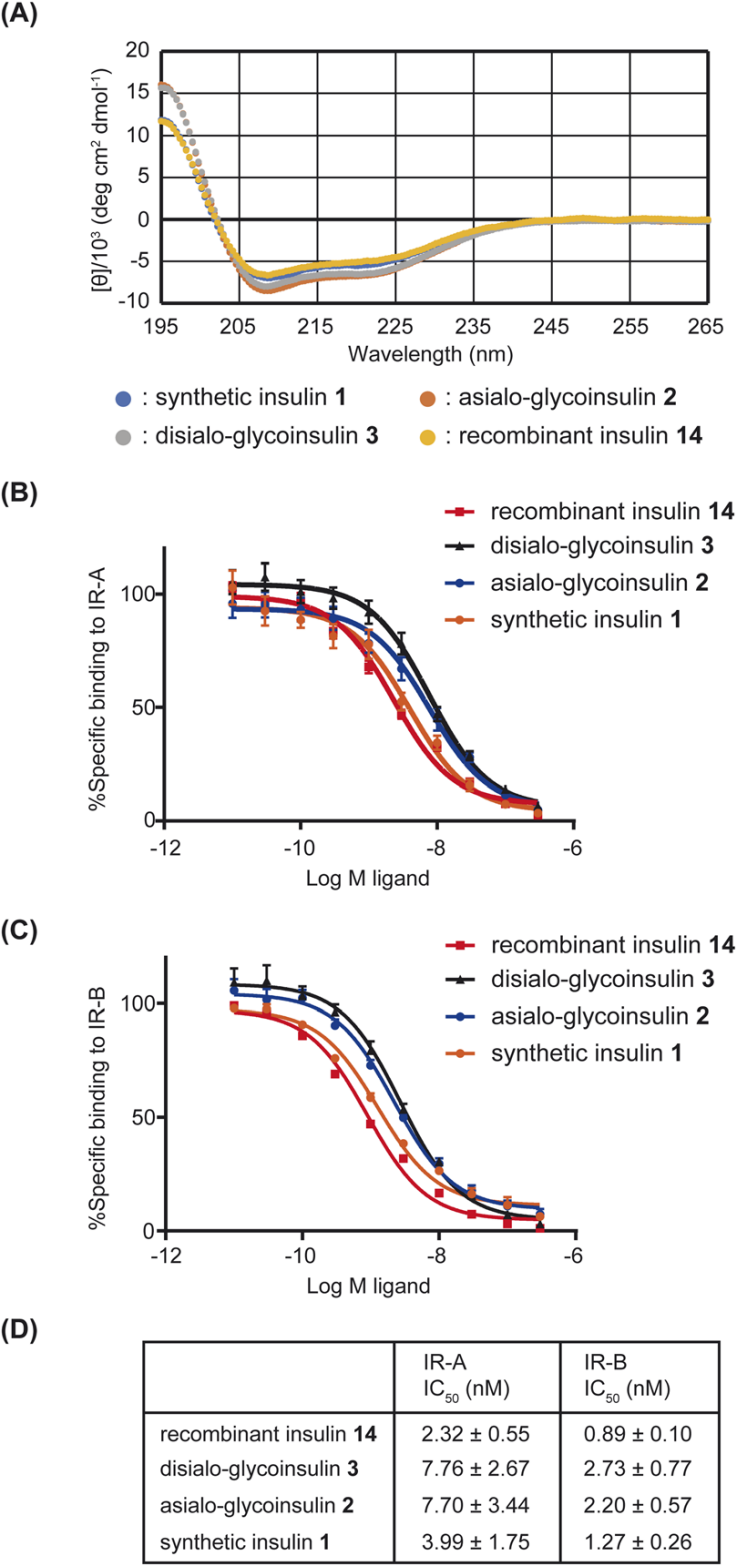
(A) UV spectra of insulin derivatives. (B) Binding assay with IR-A. (C) Binding assay with IR-B. (D) Summary of binding affinities (*n* = 3).

With synthetic native insulin 1, asialo-glycoinsulin 2, and disialo-glycoinsulin 3 in hand, we measured their far UV circular dichroism (CD) spectra. The CD patterns of insulin 1 and glycoinsulins 2 and 3 were compared with that of recombinant insulin 14. All insulin derivatives exhibited similar patterns, as shown in Fig. 8A, although slightly deeper absorbance was observed for disialo- and asialo-glycoinsulins. These data indicated that the additional glycans did not significantly alter the secondary structures of insulin, consistent with previous reports.^10*e*^

Finally, the binding ability of the insulin derivatives to insulin receptor type A (IR-A) and type B (IR-B) was measured. As previously reported,^10*e*^ we performed the competitive assay using europium-labeled insulin. The obtained IC_50_ values are summarized in Fig. 8D. Asialo- and disialo-glycoinsulins exhibited slightly reduced binding ability; however, no significant decrease was observed due to the presence of bulky glycans. These findings are consistent with the previous report.^10*e*^

## Conclusions

In summary, we utilized manual flow-based Fmoc-SPPS to synthesize glycosylated insulin derivatives. In addition to native insulin 1, we successfully obtained asialo-glycoinsulin 2 and disialo-glycoinsulin 3, which feature different types of human glycans at the N-terminal of the B-chain *via* asparagine side-chain linkage for the first time. These glycoinsulins exhibited suitable binding affinities toward IR-A and IR-B.

We also demonstrated the utility of isoacyl and pseudoproline dipeptides in combination with flow-based SPPS. In particular, the pseudoproline was compatible with the fully flow-based synthesis of the B-chain.

Compared to the reported glycoinsulin with a glycan linked *via* Cys thioether linkage,^10*e*^ our flow-based approach enabled a rapid glycosylation step (within 10 minutes) through simple amide bond formation. We envisage that the native asparagine linkage could potentially avoid immunogenicity issues. Overall, this new flow-based glycosylation method represents a promising approach to improve physicochemical properties and biological activity of peptides and proteins.

Although global yields of insulin 1 and asialo-glycoinsulin 2 (1.5% and 2.1%, respectively) were not as high as those previously reported using a standard automated peptide synthesizer,^8*b*^ we successfully demonstrated an alternative approach to rapidly access the biologically important molecules. Since manual flow synthesis does not require expensive systems and can be performed with simple equipment such as a flow vessel and an HPLC pump, our strategy is accessible rather than commercial fully automated peptide synthesizers, such as microwave-assisted synthesizer that also enables fast peptide synthesis. For further improvement of yields, an automated flow-peptide synthesizer (AFPS)^21^ would be useful to precisely optimizing the synthesis of A- and B-chain peptides.

Regarding the coupling of glycosyl-asparagine, reactions had to be repeated for completion due to the low solubility and reactivity. Our group is currently working on improving this reaction by modifying the structure of glycosyl-asparagine. Additionally, efforts are underway to incorporate glycosyl-asparagine into the native consensus sequence (Asn-X-Ser/Thr) of peptides using the flow system.

## Data availability

The data supporting this article, including synthesis, characterizations, and assays, have been included as part of the ESI.†

## Author contributions

Y. M., Y. K., and B. L. P. conceptualized the research. Y. M., S. K. M., S. Y. and C. M. F. optimized the synthesis conditions of native insulin. Y. M. synthesized, purified, and analyzed the glycoinsulin samples. B. E. F., C. C. and A. H. performed the insulin binding assay. Y. M., C. C., B. E. F., A. H., Y. K., and B. L. P. wrote the manuscript with input from all coauthors.

## Conflicts of interest

There are no conflicts to declare.

## Supplementary Material

SC-OLF-D5SC01670C-s001
