# Supplementary material for: Rapid synthesis of glycosylated insulins by flow-based peptide synthesis
Source: Chem Sci. 2025 Apr 4;16(18):7929–35. doi: 10.1039/d5sc01670c (PMC11969719; doi:10.1039/d5sc01670c)
Supplement: SC-016-D5SC01670C-s001 [file SC-016-D5SC01670C-s001.pdf]

Supplementary Information (SI) for Chemical Science.
This journal is © The Royal Society of Chemistry 2025

Electronic Supporting Information for

Rapid Synthesis of glycosylated insulins by flow-based peptide synthesis

Yuta Maki,[a,b,c*] Surin K. Mong,[c] Chaitra Chandrashekar,[d] Briony E. Forbes,[e] Mohammed Akhter Hossain,[d] Shintaro Yamaguchi,[a] Colin M. Fadzen,[c] Yasuhiro Kajihara,[a,b] Bradley L. Pentelute[c,f,g,h*]

a. Department of Chemistry, Graduate School of Science, Osaka University, 1-1 Machikaneyama, Toyonaka, Osaka 560-0043, Japan.
b. Forefront Research Center, Graduate School of Science, Osaka University, 1-1 Machikaneyama, Toyonaka, Osaka 560-0043, Japan.
c. Department of Chemistry, Massachusetts Institute of Technology, Cambridge, Massachusetts 02139, United States
d. The Florey Institute of Neuroscience and Mental Health, The University of Melbourne, Victoria 3010, Australia.
e. Discipline of Medical Biochemistry, College of Medicine and Public Health, Flinders University, Bedford Park, South Australia 5042, Australia.
f. The Koch Institute for Integrative Cancer Research, Massachusetts Institute of Technology, Cambridge, Massachusetts 02142, United States.
g. Center for Environmental Health Sciences, Massachusetts Institute of Technology, Cambridge, Massachusetts 02139, United States.
h. Broad Institute of MIT and Harvard, Cambridge, Massachusetts 02142, United States.

[*] Correspondence to: makiyt11@chem.sci.osaka-u.ac.jp, blp@mit.edu

Table of Contents:

1. Methods ……………………………………………………………………………………S3

1-1. Materials and abbreviation……………………………………………………………S3

1-2. Analytical methods……………..…………………………………...…………………S3

1-3. General condition for peptide synthesis …………………………………………………S4

2. Experimental procedures and characterizations

2-1. Synthesis of A-chain……..……………………………………..……….…………..…S5

2-2. Synthesis of B-chain …………..…………………….............................…………..…S6

2-3. Synthesis of B-chain by fully flow synthesis……………………………......…………..…S7

2-4. Optimization of peptide elongation …………..……………………...…....…………..…S8

2-5. Synthesis of glycosylated B-chain…………..……....……………………………………S9

2-6. Synthesis of glycosylated B-chain by flow condition…....……………………………………S10

2-7. Synthesis of glycosylated B-chain by flow condition ….……..……....……………………S11

2-8. Investigation on coupling of Fmoc-Asn(glycan)-OH………………………………………S13

2-9. NMR data of glycosylated B-chain……....……………………………...………………S13

2-10. Synthesis of disialylated B-chain………..……...……………………..………………S14

2-11. Folding for native insulin…………...………….………………………………….……...S15

2-12. Folding for asialo-glycoinsulin…………….……………………………………….…......S16

2-13. Synthesis of disialo-glycoinsulin………...……………...……………………….…...S17

2-14. Enzymatic synthesis of disialo-glycoinsulin…...……………………………………………S19

2-15. Spectroscopic characterization (CD spectra) …………….……….…………………………S19

2-16. Determination of disulfide linkages of native insulin ……...…………….……….………..….S20

2-17. Insulin receptor binding assay………….…………………………….…………..…..........S25

3. References………………………………………………………………………..…………S26

1 Methods

1-1 Materials and abbreviation

1-((Dimethylamino)(dimethyliminio)methyl)-1H-benzo[*d*][1,2,3]triazole 3-oxide hexafluorophosphate (HBTU), 1*H*-Benzotriazol-1-yloxytripyrrolidinophosphonium hexafluorophosphate (PyBOP), Fmoc-His(Boc)-OH, Fmoc-Arg(Pbf)-OH, Rink amide resin, and Fmoc-Cys(Mmt)-OH were purchased from WATANABE CHEMICAL INDUSTRIES, LTD (Japan). 1-Hydroxybenzotriazole (HOBt) was purchased from PEPTIDE INSTITUDE. INC (Japan). Triisopropylsilane (TIPS), N,N-diisopropylethylamine (DIEA), and 2,2'-dipyridyl disulfide were purchased from TOKYO CHEMICAL INDUSTRY CO., LTD (Japan). Dimethyl sulfoxide (DMSO), 2-amino-2-hydroxymethyl-1,3- propanediol (Tris), guanidine hydrochloride (Gn-HCl), trifluoroacetic acid (TFA), sodium chloride (NaCl), 1-methyl-2-pyrrolidone (NMP), and disodium hydrogenphosphate dodecahydrate were purchased from Wako Chemical Industries, Ltd (Japan). Amino PEGA resin, Fmoc-Leu-OH, Fmoc-Ile-OH, Fmoc-Phe-OH, Fmoc-Glu(OtBu)-OH, Fmoc-Ala-OH, Fmoc-Cys(Acm)-OH, Fmoc-Tyr(tBu)-OH, Fmoc-Val-OH, Fmoc-Ser(tBu)-OH, Fmoc-Pro-OH, Fmoc-Gly-OH, Fmoc-Thr(tBu)-OH, Fmoc-Cys(Trt)-OH, Fmoc-Asn(Trt)-OH, Fmoc-Gln(Trt)-OH, 4-hydroxymethylphenoxyacetic acid (HMPA), 1-(mesitylene-2-sulfonyl)-3-nitro-1H-1,2,4-triazole (MSNT), Fmoc-Gly-Ser(psiMe,Mepro)-OH, Fmoc-Lys(Boc)-OH, Fmoc-Cys(StBu)-OH, and 1-[Bis(dimethylamino)methylene]-1*H*-1,2,3-triazolo[4,5-*b*]pyridinium 3-oxide hexafluorophosphate (HATU) were purchased from Novabiochem. Aminomethyl ChemMatrix® resin, N,N'-diisopropylcarbodiimide (DIC), 2,2'-dithiobis(5-nitropyridine) (DTNP), and Fmoc-Asp-OtBu were purchased from SIGMA-ALDRICH. Acetonitrile (CH_3CN), *N,N'*-dimethylformamide (DMF), dichloromethane (DCM) were purchased from KANTO CHEMICAL CO., INC (Japan). Boc-Ser[Fmoc-Thr(tBu)]-OH was purchased from AAPPTEC. 1-[Bis(dimethylamino)methylene]-5-chloro-1*H*-benzotriazolium 3-oxide hexafluorophosphate (HCTU) was purchased from PEPTIDE INSTITUTE, INC (Japan).

1-2 Analytical methods

High-resolution mass spectrometry was performed by FTICR (Bruker solariX XR), Q-TOF mass spectrometry (Bruker Compact), or linear ion trap Orbitrap (Thermo Fisher Scientific, LTQ Orbitrap XL). Other mass spectra were recorded on a Bruker esquire 3000plus (ion trap), or an amaZon ETD (ion trap) mass spectrometer.

The purity and mass of synthetic peptides were confirmed by analytical liquid chromatography-mass spectrometry (LCMS) or separate LC and direct infusion.

Chromatographic separation was performed by using a linear gradient of solvent B in solvent A (solvent A: 0.1% aq TFA, solvent B: 90% aq CH_3CN containing 0.1% TFA) on a Waters 2489 system equipped with a variable wavelength detector or performed by using a linear gradient of solvent D in solvent C over 10 min (solvent C: 0.1% aq HCOOH, solvent D: 90% aq CH_3CN containing 0.09% HCOOH) for LCMS on a DIONEX UltiMate 3000 UHPLC system equipped with a variable wavelength detector. C4 silica gel (Proteonavi, Osaka soda, 4.6 mm × 250 mm) was used for analytical column. The eluent was monitored by UV absorbance at 218 nm.

For semipreparative high-performance liquid chromatography (HPLC), C4 silica gel column (Proteonavi, Osaka soda, 10 mm×250 mm) was used with a linear gradient of solvent B in solvent A (solvent A: 0.1% aq TFA, solvent B: 90% aq CH_3CN containing 0.1% TFA) on a Waters 2487 HPLC system equipped with a variable wavelength detector. Fractions containing pure material were identified by LCMS or direct infusion MS analysis.

1-3 General condition for peptide synthesis

Peptides were synthesized based on the previously reported flow platform in a heated water bath.[ref.1] Unless noted, peptide elongation was performed with a 3-minute cycle, consisting of (i) coupling of Fmoc-amino acid (ca 30 sec), (ii) washing with DMF (1 min), (iii) deprotection of Fmoc group (20 sec), and (iv) washing with DMF (1 min).

(i) During the amino acid coupling, a solution of activated Fmoc-amino acid (1 mmol) in DMF (3 mL) was delivered at 6 mL/min using a syringe pump. Prior to the coupling reactions, Fmoc-amino acids were dissolved in 0.38 M HBTU/ DMF (2.5 mL), and then DIEA (500 µL, 3 mmol) was added to the solution. In the case of cysteine and histidine, less amount of DIEA (190 µL, 1.1 mmol) was used to prevent undesired epimerization reactions.

(ii) The resin was washed with DMF (20 mL) delivered at 20 mL/min over 1 min.

(iii) Fmoc group was removed with 20% (v/v) piperidine in DMF (6.7 mL) delivered at 20 mL/min over 20 sec.

(iv) The resin was washed again with DMF (20 mL) delivered at 20 mL/min over 1 min.

2 Experimental procedures and characterizations

2-1 Synthesis of insulin A-chain 5

The peptide was prepared by the flow platform at 60 °C using HATU as a coupling reagent, except Thr^8-Ser^9. Fmoc-Asp-O^{t}Bu was used as the first amino acid, and peptide elongation was performed on the Rink amide resin (262 mg, 112 μmol). In the case of Thr^8-Ser^9, Boc-Ser-(Fmoc-Thr(tBu))-OH (409 mg, 0.7 mmol) was coupled offline using HOBt (0.7 mmol) and DIC (0.67 mmol) in DMF (1.4 mL) at room temperature for 1 h.

After the synthesis, the peptide-bound resin was separated into two tubes (ca 56 μmol × 2 tubes), and the resin was used for following reaction individually. To the resin (ca 56 μmol), 25% (v/v) 2-mercaptoethanol in DMF (5 mL) was added, and the mixture was agitated at 60 °C for 45 min. Then, the resin was washed by DMF, DCM, and DMF. The treatment with 2-mercaptoethanol was repeated to ensure the removal of S^{t}Bu group of Cys^6. Next, a solution of DTNP (1.12 mmol, 20 equiv) in DCM (5 mL) was added to the resin, and the mixture was agitated for 1 h at room temperature. Then, the resin was treated with 1% TFA and 5% TIPS in DCM (5 mL) for 2 min at room temperature to selectively deprotect Mmt group of Cys^{11}. The solution was filtered, and the cocktail (1% TFA and 5% TIPS in DCM) was added again. This procedure was repetitively performed 5 times. The resulting resin was washed by DCM. Then, DCM (5 mL) was added to the resin, and the mixture was agitated for 1 h at room temperature to allow for intra-A-chain disulfide bond formation. After washing with DCM, the resin was dried in vacuo for 3 h. Then, 2.5% H_2O and 2.5% TIPS in TFA (10 mL) was added, and the mixture was kept at 60 °C for 10 min. After filtration, ice-cold diethylether was added to the filtrate, and the resulting precipitate was collected by centrifuge. The precipitate was dissolved into 50% aq CH_3CN containing 0.1% TFA and lyophilized. The residue was then purified by RP-HPLC (Proteonavi, 10×250 mm, 0.1% aq TFA: 90% aq CH_3CN containing 0.1% TFA = 85:15 to 45:55 over 60 min at 4 mL/min). Same protocol was performed using the remaining resin (ca 56 μmol) to give the desired A-chain (white solid, 10.0 mg, 4%, calculated from resin). HRMS (ESI-FTICR): *m/z* calcd for $C_{102}H_{159}N_{26}O_{36}S_4$: $[M+H]^+$ 2452.0288, found: 2452.0175.

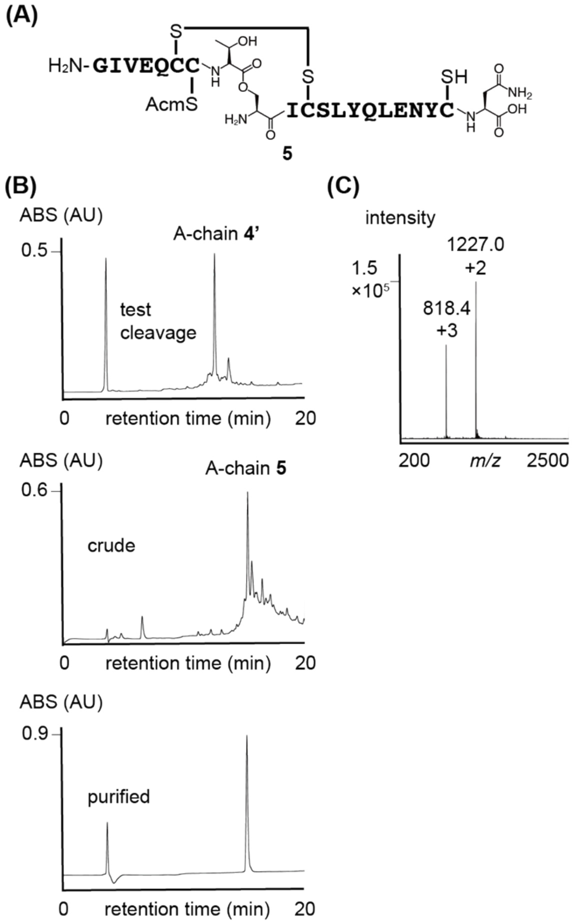

Figure S1. (A) Structure of insulin A-chain **5**. (B) HPLC analysis of peptide **4'** (test cleavage) and peptide **5**. (RT: 15.3 min, C4 column, 15% to 80% solvent B in solvent A over 20 min at 1 mL/min, absorbance at 218 nm) (C) Direct infusion ESI-MS analysis of peptide **5**. Calcd Mass (average isotope composition): $[M+H]^+$: 2453.8, $[M+2H]^{2+}$: 1227.4, $[M+3H]^{3+}$: 818.6.

2-2 Synthesis of insulin B-chain 9

The peptide assembly was performed on a 51 μmol scale by use of HMPA-ChemMatrix resin. Fmoc-Thr(^{t}Bu)-OH (5 equiv) was attached to the resin in the presence of MSNT (5 equiv) and N-methylimidazole (3.75) in DCM (1 mL) at room temperature for 2 h. The resulting resin was washed with DCM and DMF. Then, the peptide elongation was performed by the flow platform at 80 °C using HCTU as a coupling reagent except Gly^8-Ser^9. In the case of Gly^8-Ser^9, Fmoc-Gly-Ser(psiMe,MePro)-OH (0.7 mmol) was coupled offline (without flow system) using HOBt (0.67 mmol) and DIC (0.67 mmol) in DMF (1.4 mL) at room temperature for 1 h. In the case of Leu^{11}-Ala^{14}, we found that coupling reactions did not proceed efficiently compared to other amino acids (Fig. S4). Thus, the elongation cycle was extended (7 min 40 sec) as follows:

coupling of Fmoc-amino acid (ca 30 sec), (ii) dead time (flow was stopped, 3 min), (iii) washing with DMF (1 min), (iv) deprotection of Fmoc group (30 sec), (v) washing with DMF (1 min), (vi) deprotection of Fmoc group (30 sec), and (vii) washing with DMF (1 min).

After the synthesis, the peptide-bound resin was washed with DCM and dried in vacuo. To the resin, a solution of DTNP (310 mg, 1 mmol) in H_2O/ TIPS/ TFA (2.5%/ 2.5%/ 95%, 10 mL) was added. The resulting mixture was kept at room temperature for 2 h and filtered. The precipitate was obtained by the addition of ice-cold diethylether and following centrifuge. The residue was then purified by RP-HPLC (Proteonavi, 10×250 mm, 0.1% aq TFA: 90% aq CH_3CN containing 0.1% TFA = 70:30 to 40:60 over 60 min at 4 mL/min) to give the desired B-chain (white solid, 4.5 mg, 2%, calculated from resin). HRMS (ESI-FTICR): *m/z* calcd for $C_{166}H_{242}N_{43}O_{45}S_3$: $[M+H]^+$ 3653.7127, found: 3653.7005.

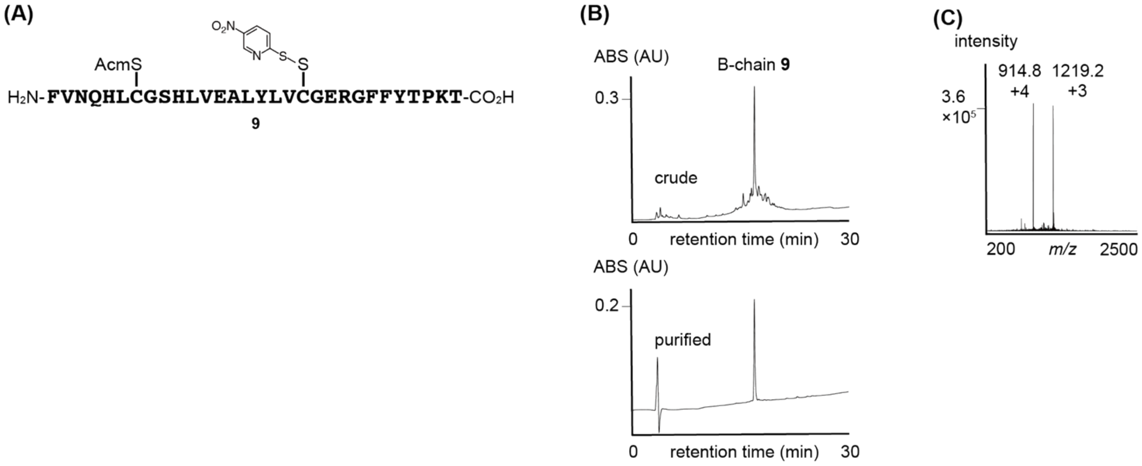

Figure S2. (A) Structure of insulin B-chain **9**. (B) HPLC analysis of peptide **9**. (RT: 16.9 min, C4 column, 30% to 90% solvent B in solvent A over 30 min at 1 mL/min, absorbance at 218 nm) (C) Direct infusion ESI-MS analysis of peptide **9**. Calcd Mass (average isotope composition): $[M+H]^+$: 3656.2, $[M+3H]^{3+}$: 1219.4, $[M+4H]^{4+}$: 914.8.

2-3 Synthesis of insulin B-chain 9 by fully flow synthesis

The peptide assembly was performed on a 20 μmol scale by use of HMPA-ChemMatrix resin with a same procedure as above (2-2) except Gly^8-Ser^9. Fmoc-Gly-Ser(psiMe,MePro)-OH (1 mmol) was dissolved in 0.38 M HBTU in DMF. After addition of DIEA (500 μL, 2.9 mmol), the dipeptide unit was coupled with a standard 3-min cycle in the flow system.

Yield: 6.31 mg, 10% (from the loading of Fmoc-Thr^{B30}). HRMS (ESI-QTOF): *m/z* calcd for $C_{166}H_{242}N_{43}O_{45}S_3$: $[M+H]^+$ 3653.7127, found: 3653.6854.

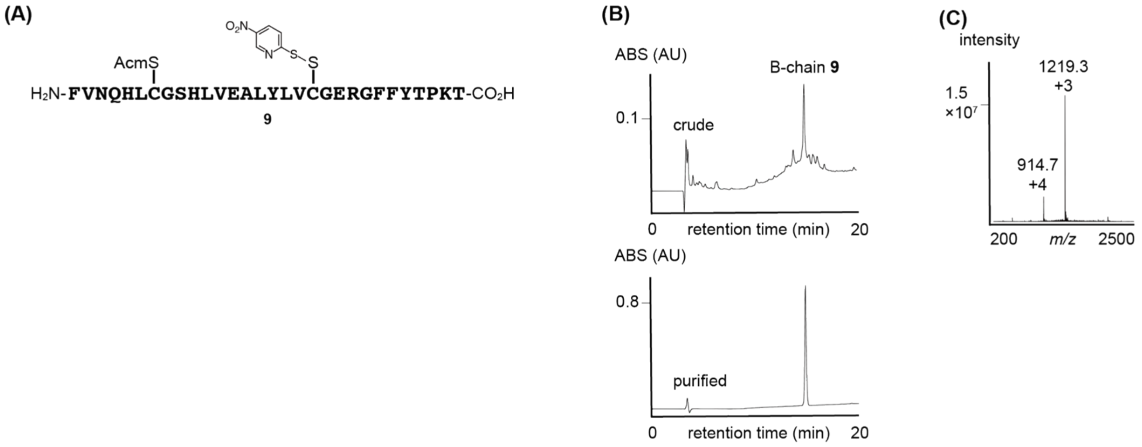

Figure S3. (A) Structure of insulin B-chain **9**. (B) HPLC analysis of peptide **9**. (RT: 14.9 min, C4 column, 30% to 90% solvent B in solvent A over 20 min at 1 mL/min, absorbance at 218 nm) (C) Direct infusion ESI-MS analysis of peptide **9**. Calcd Mass (average isotope composition): $[M+H]^{+}$: 3656.2, $[M+3H]^{3+}$: 1219.4, $[M+4H]^{4+}$: 914.8.

2-4 Optimization of peptide elongation

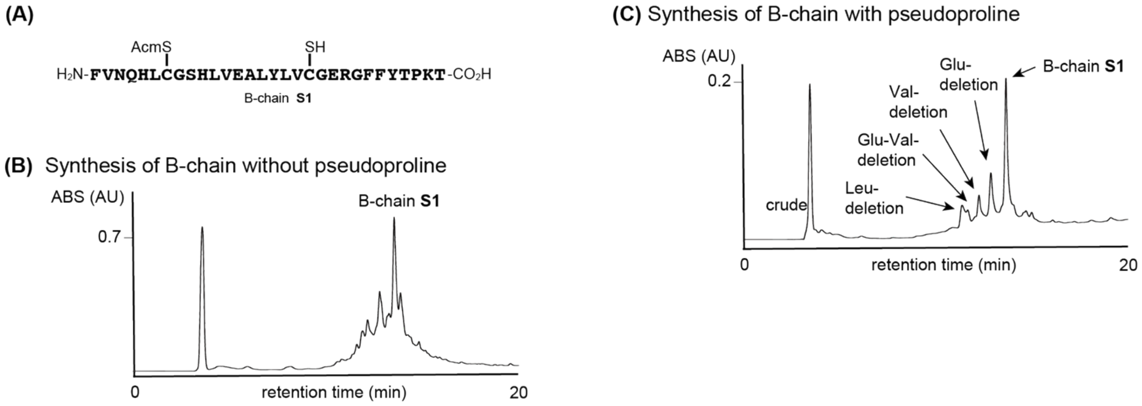

Figure S4. Representative data for optimization of peptide elongation. (A) A structure of B-chain derivative **S1**. (B) Analytical LC data for the initial synthesis of B-chain. Synthetic condition: standard 3-min cycle, 60 °C, 0.38 M HBTU, Cys^{B7}(Acm) and Cys^{B19}(Trt) were used. The pseudoproline dipeptide was not used. Based on HPLC and LCMS analyses, we found deletion of amino acids in the N-terminal region. (C) Analytical LC data for B-chain without extended coupling and deprotection for Leu^{11}, Val^{12}, Glu^{13}, and Ala^{14}. Synthetic condition: standard 3-min cycle, 80 °C, 0.38 M HBTU, Fmoc-Gly-Ser(psiMe,MePro)-OH was introduced to Gly^{8}-Ser^{9}, the Acm and Trt groups were used for Cys^{7} and Cys^{19}, respectively.

2-5 Synthesis of glycosylated insulin B-chain 12 (Entry 3)

The peptide assembly was performed on a 33 μmol scale by use of HMPA-PEGA resin. To the resin, Fmoc-Thr(^{t}Bu)-OH (99.4 mg, 0.25 mmol), MSNT (74.1 mg, 0.25 mmol), and N-methylimidazole (14.8 μL, 0.19 mmol) in DCM (1 mL) were added, and the mixture was agitated at room temperature for 2 h. The resulting resin was washed with DCM and DMF. Then, the peptide elongation was performed by the flow platform at 80 °C using HATU as a coupling reagent except Gly^8-Ser^9. In the case of Gly^8-Ser^9, Fmoc-Gly-Ser(psiMe,MePro)-OH (0.7 mmol) was coupled offline (without flow system) using HOBt (0.67 mmol) and DIC (0.67 mmol) in DMF (1.4 mL) at room temperature for 1 h. In the case of Leu^{11}-Ala^{14}, the elongation cycle was extended (7 min 40 sec) as follows: coupling of Fmoc-amino acid (ca 30 sec), (ii) dead time (flow was stopped for 3 min), (iii) washing with DMF (1 min), (iv) deprotection of Fmoc group (30 sec), (v) washing with DMF (1 min), (vi) deprotection of Fmoc group (30 sec), and (vii) washing with DMF (1 min).

After the synthesis, Fmoc-Asn(asialooligosacchairde)-OH **11** (91.9 mg, 46.5 μmol), which was prepared from egg yolk,[ref.2] PyBOP (34.2 mg, 65.7 μmol), and DIEA (40.8 μL, 0.24 mmol) in NMP/ DMSO (1.5 mL/1.5 mL) were added to the resin, and the mixture was agitated at room temperature in dark for 13 h. The resulting resin was then washed with DMF. To the resin, 20% piperidine in DMF (2 mL) was added and the mixture was agitated at room temperature for 20 min. The resulting resin was washed with DMF and DCM. To the resin, a solution of DTNP (376 mg, 1.21 mmol) in water/ TIPS/ TFA (2.5%/ 2.5%/ 95%, 5 mL) was added. The resulting mixture was kept at room temperature for 2 h and filtered. The precipitate was obtained by the addition of ice-cold diethylether and following centrifuge. The residue was then purified by RP-HPLC (Proteonavi, 10×250 mm, 0.1% aq TFA: 90% aq CH_3CN containing 0.1% TFA = 75:25 to 50:50 over 60 min at 4 mL/min) to give the desired B-chain (white solid, 6.1 mg, 4%, calculated from resin). HRMS (ESI-FTICR): *m/z* calcd for $C_{232}H_{350}N_{49}O_{92}S_3$: $[M+H]^+$ 5390.3372, found: 5390.3331.

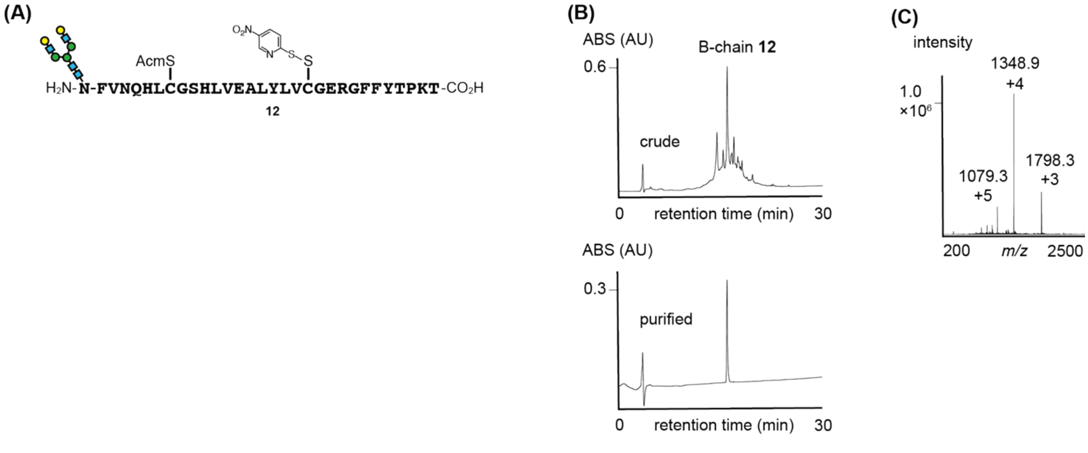

Figure S5. (A) Structure of glycosylated B-chain **12**. (B) HPLC analysis of glycopeptide **12**. (RT: 16.0 min, C4 column, 30% to 90% solvent B in solvent A over 30 min at 1 mL/min, absorbance at 218 nm) (C) Direct infusion ESI-MS analysis of glycopeptide **12**. Calcd Mass (average isotope composition): $[M+H]^{+}$: 5393.8, $[M+3H]^{3+}$: 1798.6, $[M+4H]^{4+}$: 1349.2, $[M+5H]^{5+}$: 1079.6.

2-6 Synthesis of glycosylated insulin B-chain 12 by flow condition (Entry 2)

The peptide assembly was performed on a HMPA-PEGA resin with a similar manner as above (2-4) except coupling of Fmoc-Asn(asialooligosaccharide)-OH **11**. After the flow-based coupling of Phe^{B1}, Fmoc-Asn(asialooligosaccharide)-OH (33 mM) was activated by PyBOP (67 mM) and DIEA (67 mM) in NMP-DMSO (2:1, 2.5 mL), and the resulting solution was delivered to the resin in the flow reactor by the syringe pump. The reactor was left at rt for 15 min. This coupling step was repeated (double coupling in total). Then, N-terminal Fmoc group was removed under the flow condition. The resulting resin was subjected to the global deprotection steps and HPLC purification as mentioned above. Yield: 6.6 mg (isolated yield 8% from the loading of Fmoc-Thr^{B30}), white solid. HRMS (ESI-QTOF): *m/z* calcd for $C_{232}H_{350}N_{49}O_{92}S_3$: $[M+H]^{+}$ 5390.3372, found: 5390.2955.

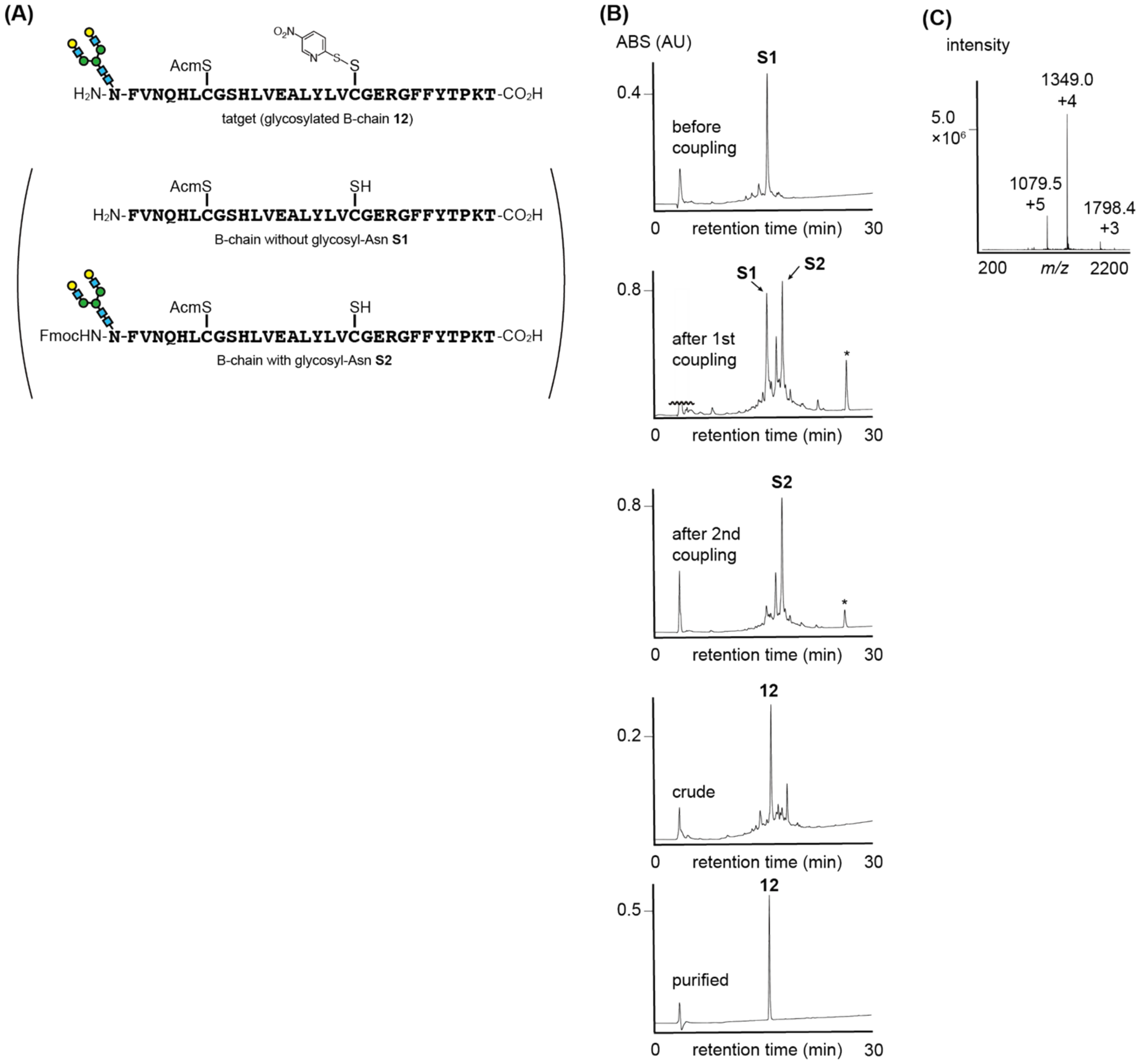

Figure S6. (A) Structures of B-chain derivatives. (B) HPLC analysis of glycopeptide **12**. (RT: 16.0 min, C4 column, 30% to 90% solvent B in solvent A over 30 min at 1 mL/min, absorbance at 218 nm) (C) Direct infusion ESI-MS analysis of glycopeptide **12**. Calcd Mass (average isotope composition): $[M+H]^{+}$: 5393.8, $[M+3H]^{3+}$: 1798.6, $[M+4H]^{4+}$: 1349.2, $[M+5H]^{5+}$: 1079.6.

2-7 Synthesis of glycosylated insulin B-chain 12 by flow condition (Entry 1)

The peptide assembly was performed on a HMPA-PEGA resin with a similar manner as above (2-4) except coupling of Fmoc-Asn(asialooligosaccharide)-OH **11**. After the flow-based coupling of Phe^{B1}, Fmoc-Asn(asialoglycan)-OH (33 mM) was activated by PyBOP (67 mM) and DIEA (67 mM) in NMP-DMSO (2:1, 2.5 mL), and the resulting solution was delivered to the resin in the flow reactor by the syringe pump at 60 ^{o}C. The reactor was left at 60 ^{o}C for 3 min. This coupling step was repeated twice (triple coupling in total). Then, N-terminal Fmoc group

was removed under the flow condition. The resulting resin was subjected to the global deprotection steps and HPLC purification as mentioned above. Yield: 12.2 mg, (isolated yield 11% from the loading of Fmoc-Thr^{B30}), white solid. HRMS (ESI-QTOF): *m/z* calcd for $C_{232}H_{350}N_{49}O_{92}S_3$: $[M+H]^+$ 5390.3372, found: 5390.3142.

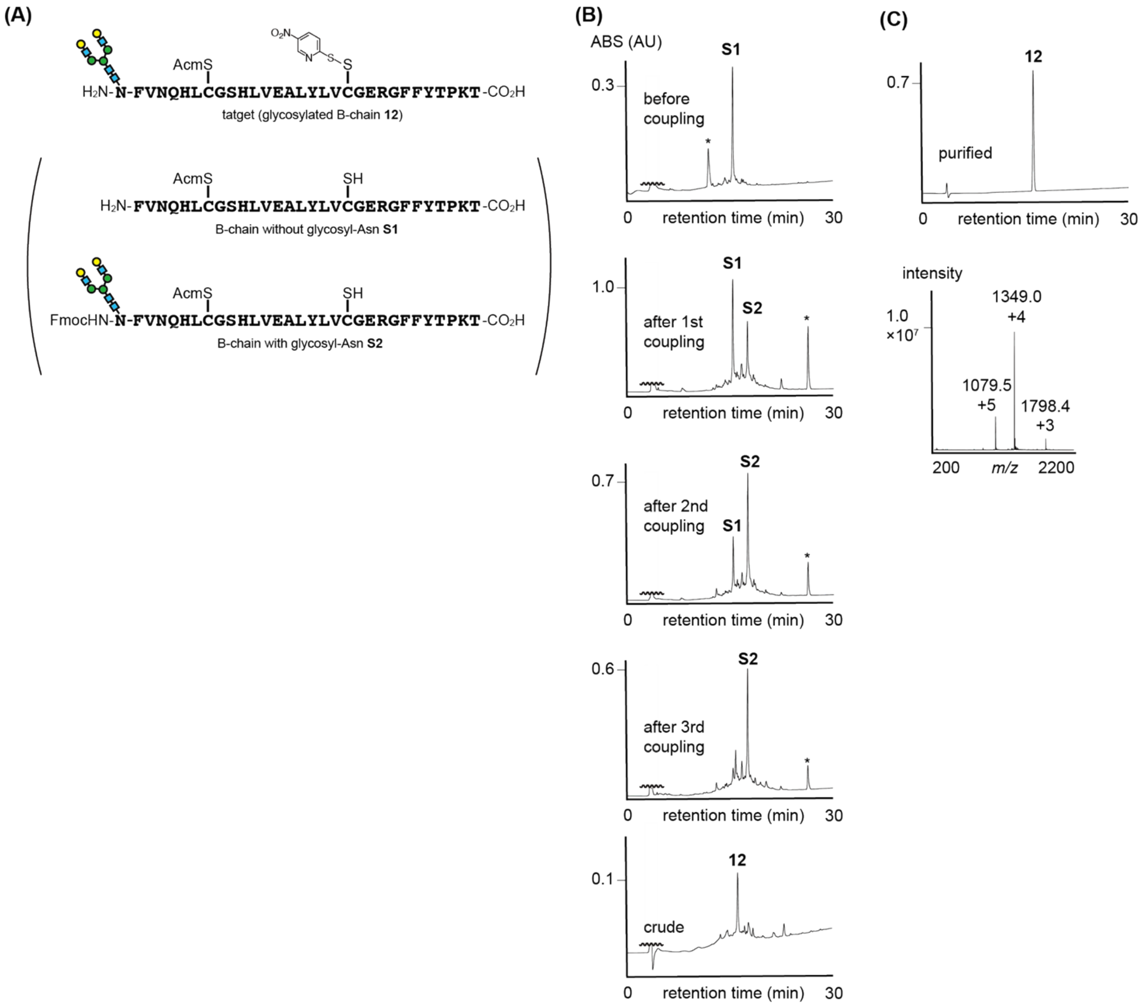

Figure S7. (A) Structures of B-chain derivatives. (B) HPLC analysis of glycopeptide **12**. (RT: 16.0 min, C4 column, 30% to 90% solvent B in solvent A over 30 min at 1 mL/min, absorbance at 218 nm) (C) Direct infusion ESI-MS analysis of glycopeptide **12**. Calcd Mass (average isotope composition): $[M+H]^+$: 5393.8, $[M+3H]^{3+}$: 1798.6, $[M+4H]^{4+}$: 1349.2, $[M+5H]^{5+}$: 1079.6. Asterisks (*) indicate non-peptidic peaks.

2-8 Investigation on coupling of Fmoc-Asn(asialooligosaccharide)-OH to the B-chain

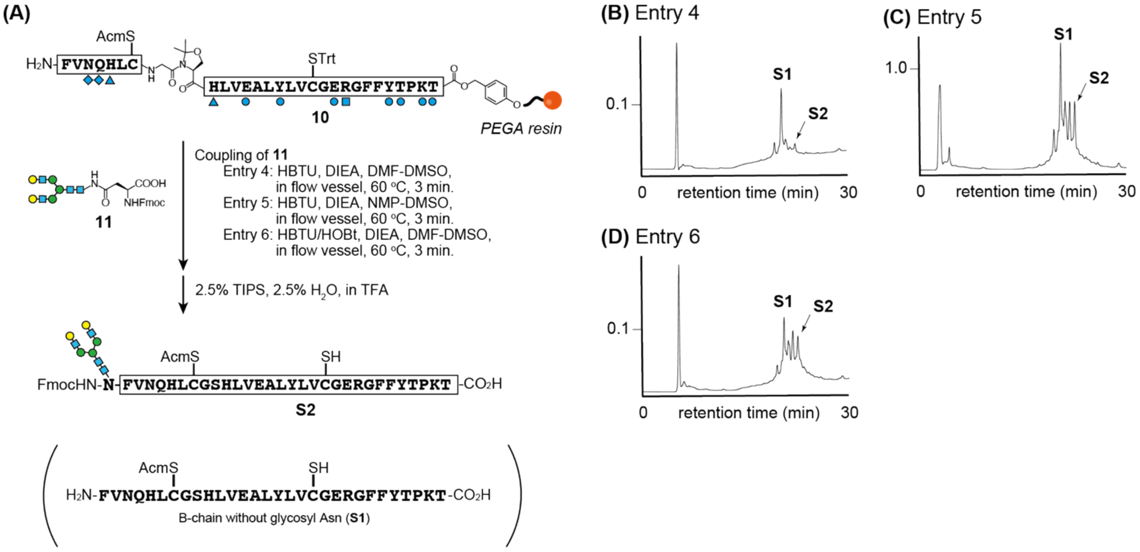

Figure S8. (A) Scheme of the coupling reaction. (B), (C), and (D) HPLC monitoring. Entry 4 (B): Fmoc-Asn(asialooligosaccharide)-OH (162 mM), HBTU (152 mM), DIEA (448 mM) in DMF-DMSO (2.0 mL/ 0.5 mL), 60 °C, 3 min in a flow reactor. Entry 5 (C): Fmoc-Asn(asialooligosaccharide)-OH (58 mM), HBTU (54 mM), DIEA (158 mM) in NMP-DMSO (2.0 mL/ 1.5 mL), 60 °C, 3 min in a flow reactor. Entry 6 (C): Fmoc-Asn(asialooligosaccharide)-OH (162 mM), HBTU/HOBt (152 mM), DIEA (448 mM) in DMF-DMSO (2.0 mL/ 1.5 mL), 60 °C, 3 min in a flow reactor. We also tested the coupling using DEPBT as an activating reagent, but the results were not successful.

2-9 NMR data of glycosylated B-chain 12

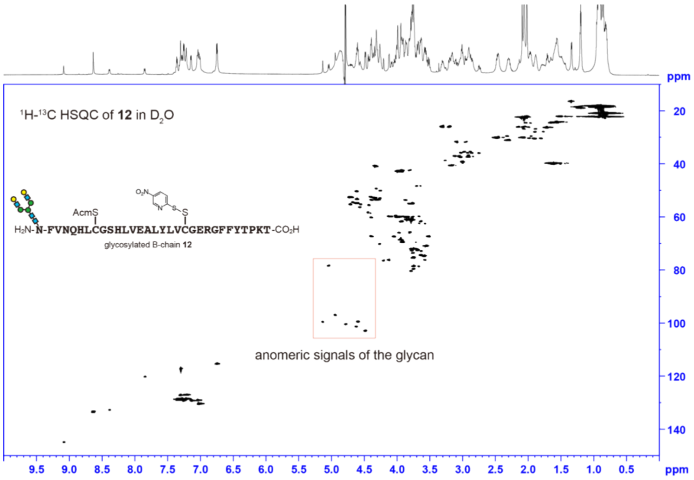

Figure S9. ^1H-^{13}C HSQC spectrum of **12**. A region of anomeric signals of asialo-glycan is highlighted. Based on these anomeric signals, we concluded that we indeed obtained single

isomer.

2-10 Synthesis of disialylated B-chain 13

The peptide elongation was performed in the similar manner as glycosylated B-chain (2-5). Briefly, Fmoc-Thr(^{t}Bu)-OH was coupled to HMPA-PEGA resin (10 μmol) and then peptide elongation was performed by the flow platform at 70 °C using HBTU as a coupling reagent. Fmoc-Gly-Ser(psiMe,MePro)-OH was introduced under the flow condition. In the case of Leu^{11}-Ala^{14}, the elongation cycle was extended (7 min 40 sec) as mentioned above. After the flow-based coupling of Phe^{B1}, Boc-Asn(diPac-disialylated oligosaccharide)-OH [ref 3] (33 mM) was activated by PyBOP (67 mM) and DIEA (67 mM) in NMP-DMSO (2:1), and the resulting solution was delivered to the resin in the flow reactor by the syringe pump. The reactor was left at rt for 15 min, and the flow was stopped only during this coupling reaction. This coupling step was repeated two times (triple coupling in total). The resulting resin was subjected to the global deprotection steps (2.5% TIPS and 2.5% H_2O in TFA), and the following lyophilization yielded crude material (23.4 mg).

An aliquot of the material (0.84 mg) was dissolved in freshly prepared 0.2 M phosphate buffer (170 μL, pH 4.2) containing 6 M Gn-HCl, and freshly prepared zinc-cooper couple (20 mg) was added to the mixture, which was agitated at room temperature for 12 h. Then, the mixture was centrifuged, and 2,2'-dipyridyl disulfide (2.0 mg, 9.1 μmol) was added to the supernatant. After 3 h, the mixture was diluted with aq CH_3CN containing 0.1 % TFA and purified by RP-HPLC (Proteonavi, 10×250 mm, 0.1% aq TFA: 90% aq CH_3CN containing 0.1% TFA = 70:30 to 40:60 over 60 min at 4 mL/min) to give the desired disialylated B-chain **11** (white solid, 0.14 mg, 0.024 umol, 7%, from the loading of Fmoc-Thr^{B30}). HRMS (ESI-QTOF): *m/z* calcd for $C_{254}H_{385}N_{50}O_{106}S_3$: $[M+H]^+$ 5927.5430, found: 5927.5216.

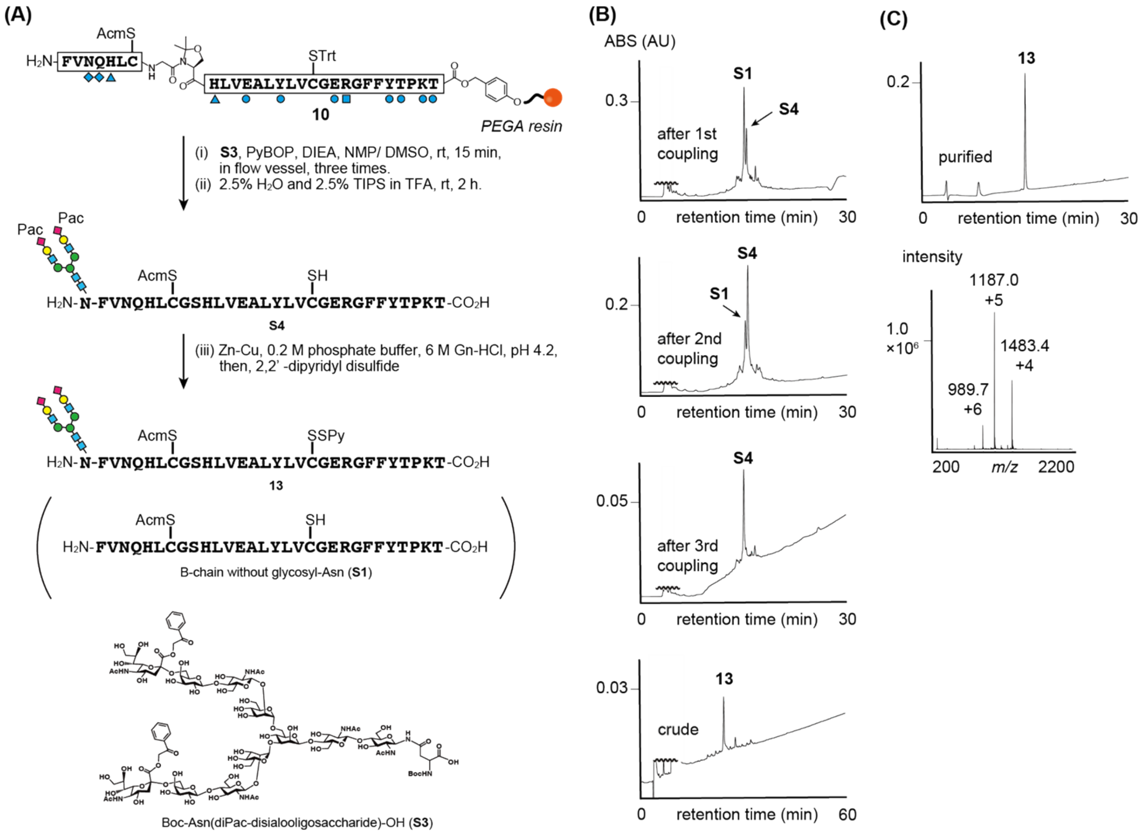

Figure S10. (A) Scheme of the synthesis of disialylated B-chain **13**. (B) HPLC analysis of glycopeptide **13**. (RT: 15.0 min, C4 column, 30% to 90% solvent B in solvent A over 30 min at 1 mL/min, absorbance at 218 nm) (C) HPLC and direct infusion ESI-MS analysis of purified glycopeptide **13**. Calcd Mass (average isotope composition): $[M+H]^+$: 5931.3, $[M+4H]^{4+}$: 1843.6, $[M+5H]^{5+}$: 1187.1, $[M+6H]^{6+}$: 989.4.

2-11 Folding for native insulin 1

A-chain (4.4 mg, 1.8 µmol) and B-chain (5.7 mg, 1.6 µmol) were dissolved in 0.2 M NH_4OAc buffer (825 µL, pH 4.5) containing 6 M Gn-HCl, and the resulting mixture was agitated at room temperature. After 3 h, 0.1 M Tris-HCl buffer (825 µL, pH 7.9) containing 6 M Gn-HCl was added to the mixture, then the pH value of the mixture was adjusted to 8.0 by the addition of 5 M aq NaOH. After 30 min, iodine (28.4 µmol) in AcOH (1.65 mL) was added to the mixture. After 30 min, 1 M ascorbic acid (360 µL) was added. The resulting mixture was diluted by distilled water (2 mL), filtered, and purified by RP-HPLC (Proteonavi, 10×250 mm, 0.1% aq TFA: 90% aq CH_3CN containing 0.1% TFA = 99:01 to 75:25 over 10 min then 75:25 to 40:60 over 30 min at 2.5 mL/min) to give folded insulin **1** (white solid, 1.5 mg, 15%, calculated from B-chain). HRMS (ESI-LTQ-Orbitrap): *m/z* calcd for $C_{257}H_{387}N_{65}O_{77}S_6$: $[M+4H]^{4+}$ 1451.9167, found:

1451.9106.

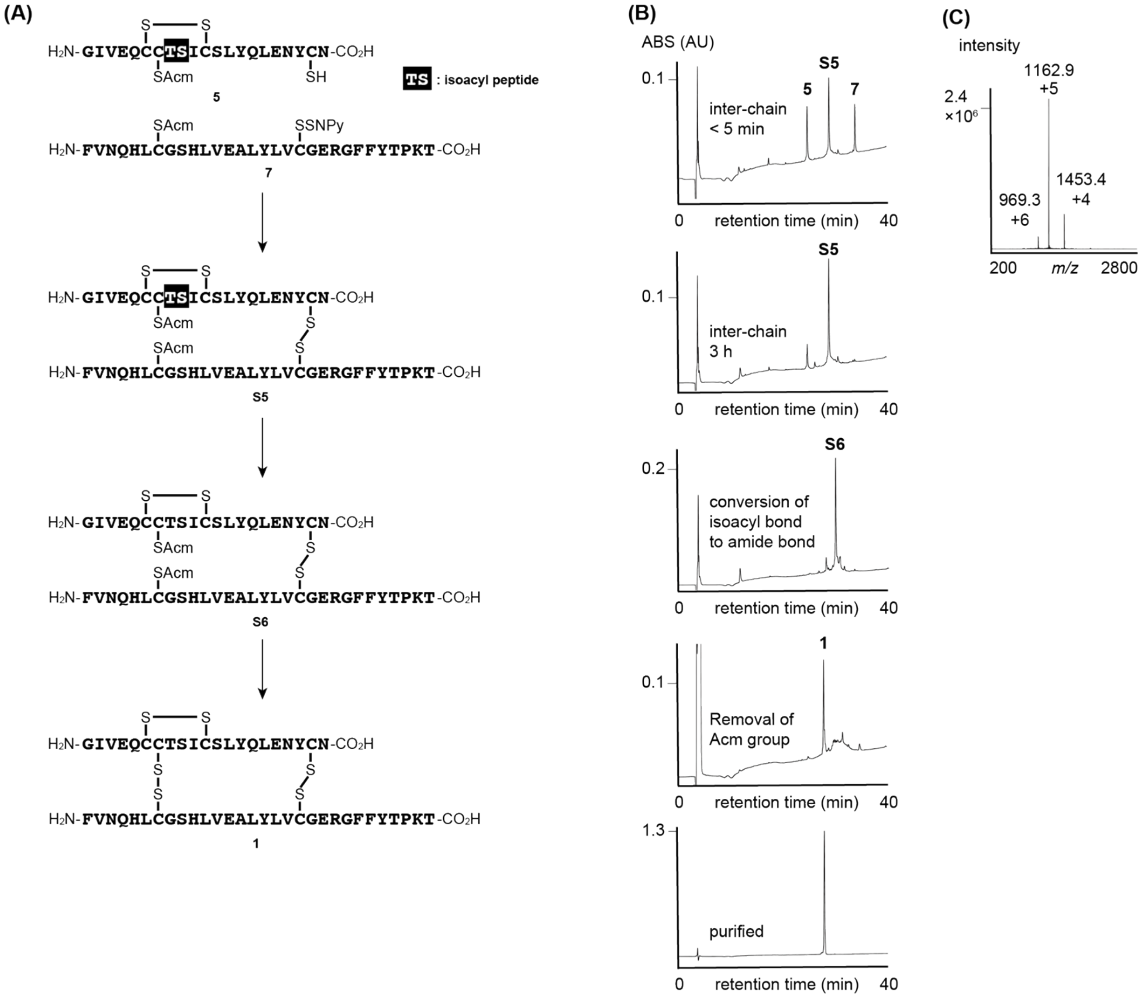

Figure S11. (A) Synthetic scheme of native insulin **1**. (B) HPLC analysis of insulin **1**. (Retention time (RT): 27.9 min, C4 column, 1% to 25% solvent B in solvent A over 10 min then 25% to 65% solvent B in solvent A over 30 min at 1 mL/min, absorbance at 218 nm) RTs of **S5** and **S6** were 28.8 and 29.9. (C) Direct infusion ESI-MS analysis of insulin **1**. Calcd Mass (average isotope composition): $[M+H]^+$: 5808.6, $[M+4H]^{4+}$: 1452.9, $[M+5H]^{5+}$: 1162.5, $[M+6H]^{6+}$: 968.9.

2-12 Folding of asialo-glycoinsulin 2

A-chain (1.0 mg, 0.41 μmol) and glycosylated B-chain (2.0 mg, 0.37 μmol) were dissolved in 0.2 M NH_4OAc buffer (185 μL, pH 4.5) containing 6 M Gn-HCl, and the resulting mixture was agitated at room temperature. After 3 h, 0.1 M Tris-HCl buffer (185 μL, pH 8.4) containing 6 M Gn-HCl was added to the mixture, then the pH value of the mixture was adjusted to 8.2 by the addition of 5 M aq NaOH. After 30 min, iodine (6.4 μmol) in AcOH (0.37 mL) was added to the mixture. After 30 min, 1 M ascorbic acid (80 μL) was added. The resulting mixture was diluted

by distilled water (0.5 mL), filtered, and purified by RP-HPLC (Proteonavi, 10×250 mm, 0.1% aq TFA: 90% aq CH_3CN containing 0.1% TFA = 99:01 to 70:30 over 10 min then 70:30 to 30:70 over 30 min at 2.5 mL/min) to give folded insulin (white solid, 0.5 mg, 19%, calculated from B-chain). HRMS (ESI-FTICR): *m/z* calcd for $C_{323}H_{492}N_{71}O_{124}S_6$: $[M+H]^+$ 7541.2695, found: 7541.2914.

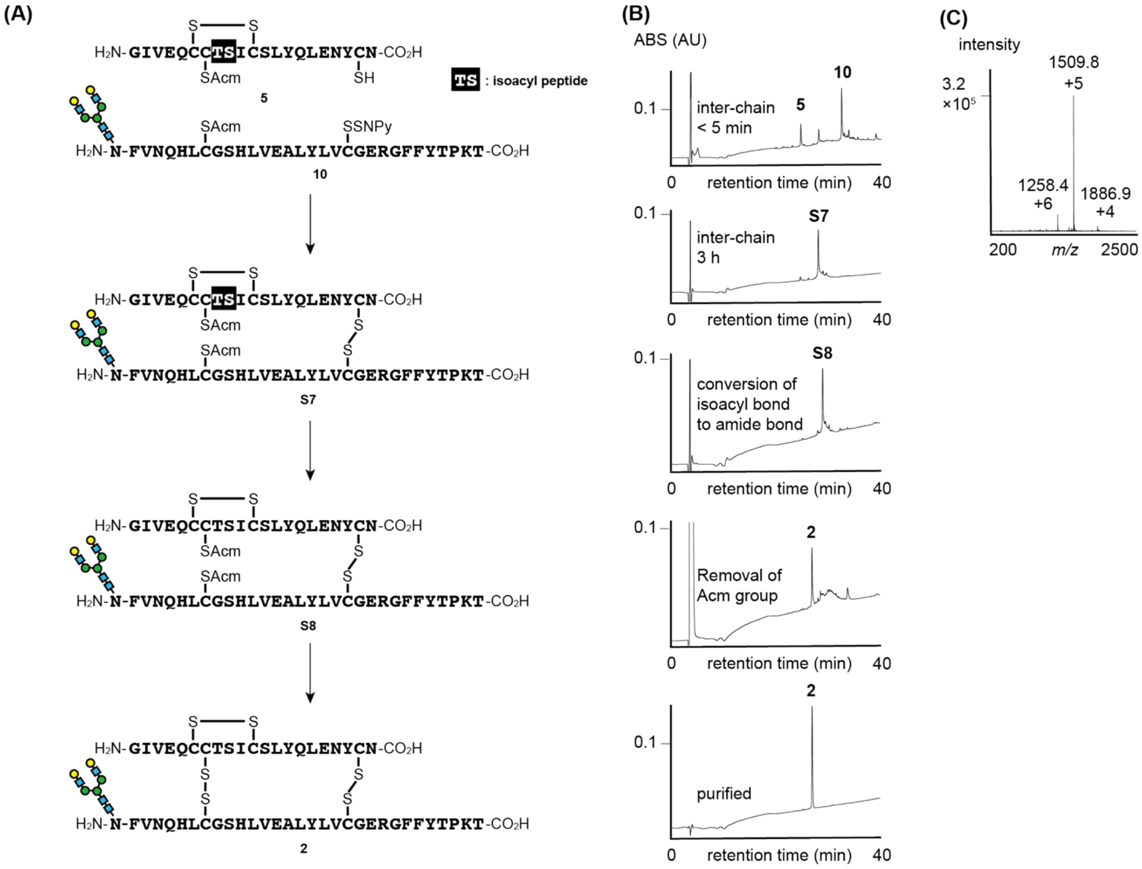

Figure S12. (A) Synthetic scheme of asialo-glycoinsulin **2**. (B) HPLC analysis of asialo-glycoinsulin **2**. (Retention time (RT): 27.0 min, C4 column, 1% to 25% solvent B in solvent A over 10 min then 25% to 65% solvent B in solvent A over 30 min at 1 mL/min, absorbance at 218 nm) RTs of **S5** and **S6** were 23.9 and 25.1. (C) Direct infusion ESI-MS analysis of asialo-glycoinsulin **2**. Calcd Mass (average isotope composition): $[M+H]^+$: 7546.2, $[M+4H]^{4+}$: 1887.3, $[M+5H]^{5+}$: 1510.1, $[M+6H]^{6+}$: 1258.5.

2-13 Folding of disialo-glycoinsulin 3

A-chain (0.16 mg, 0.065 μmol) and disialylated B-chain (0.23 mg, 0.039 μmol) were dissolved in 0.2 M NH_4OAc buffer (19.4 μL, pH 5.0) containing 6 M Gn-HCl, and the resulting mixture was agitated at room temperature. After 3 h, 0.1 M Tris-HCl buffer (31 μL, pH 8.4) containing 6 M Gn-HCl was added to the mixture, then the pH value of the mixture was adjusted

to 9.2 by the addition of 5 M aq NaOH. After 30 min, iodine (2.4 μmol) in AcOH (50 μL) was added to the mixture. After 30 min, 1 M ascorbic acid (20 μL) was added. The resulting mixture was diluted by distilled water, filtered, and purified by RP-HPLC (Proteonavi, 4.6×250 mm, 0.1% aq TFA: 90% aq CH_3CN containing 0.1% TFA = 99:01 to 70:30 over 10 min then 70:30 to 30:70 over 30 min at 1.0 mL/min) to give folded insulin (white solid, 0.08 mg, 20%, calculated from B-chain). HRMS (ESI-QTOF): *m/z* calcd for $C_{345}H_{530}N_{73}O_{140}S_6$: $[M+5H]^{5+}$ 1626.5003 (most intense peak), found: 1626.4941.

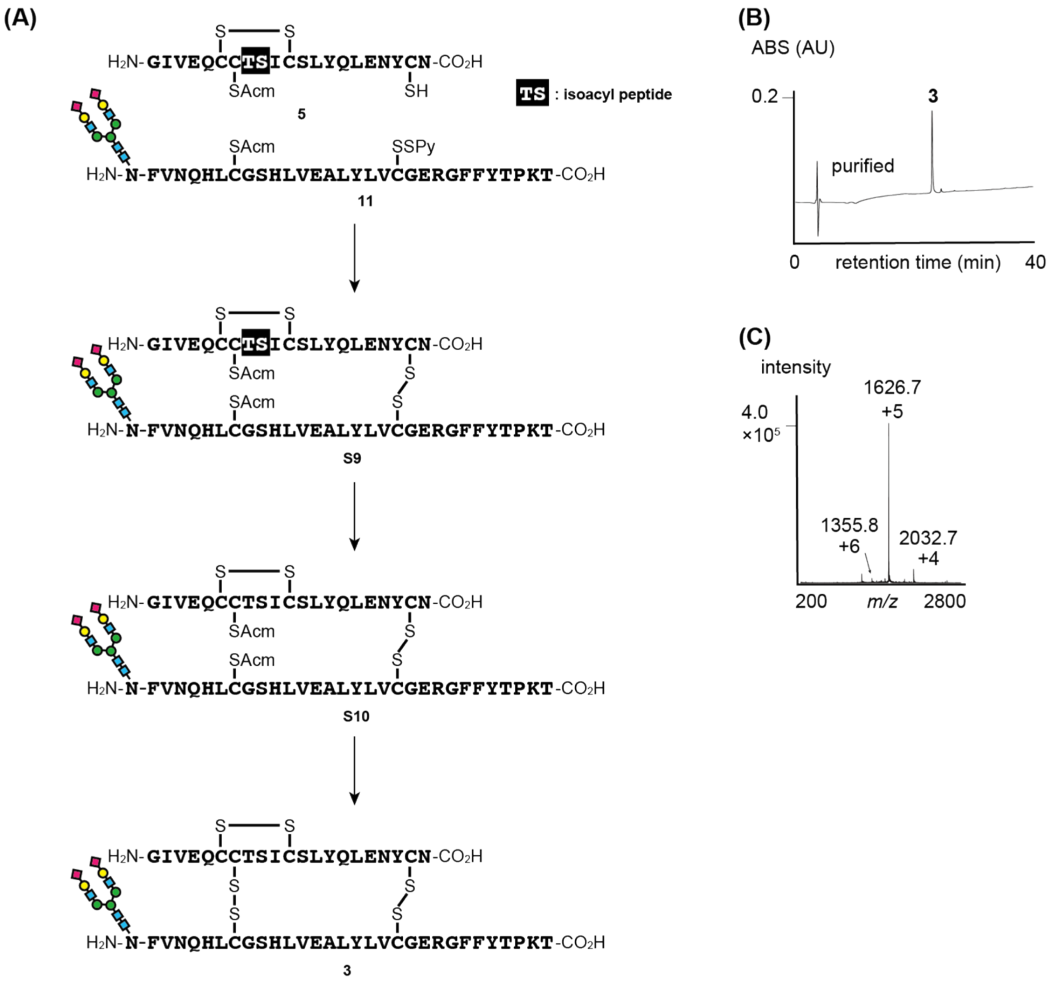

Figure S13. (A) Synthetic scheme of disialo-glycoinsulin **3**. (B) HPLC analysis of purified disialo-glycoinsulin **3**. (RT: 23.0 min, C4 column, 1% to 30% solvent B in solvent A over 10 min then 30% to 70% solvent B in solvent A over 30 min at 1 mL/min, absorbance at 218 nm) (C) Direct infusion ESI-MS analysis of disialo-glycoinsulin **3**. Calcd Mass (average isotope composition): $[M+H]^{+}$: 8128.8, $[M+4H]^{4+}$: 2032.9, $[M+5H]^{5+}$: 1626.5, $[M+6H]^{6+}$: 1355.6.

2-14 Enzymatic synthesis of disialo-glycoinsulin 3

Asialo-glycoinsulin **2** (0.9 mg, 0.12 μmol, final: 1 mM) and CMP-Neu5Ac (0.74 mg, 1.2 μmol) were dissolved in 50 mM HEPES buffer (pH 6.0, 116 μL) containing BSA (0.1 mg/mL), alkaline phosphatase (Calf intestine, 0.4 U), and α2,6-sialyltransferase (9.1 U, *P. damsela*).[ref.4] The resulting mixture was incubated at room temperature for 22 h, and then purified by RP-HPLC (Proteonavi, 4.6×250 mm, 0.1% aq TFA: 90% aq CH_3CN containing 0.1% TFA = 99:01 to 80:20 over 10 min then 80:20 to 40:60 over 30 min at 1 mL/min) to give disialo-glycoinsulin (white solid, 0.8 mg, 0.10 umol, 84%). HRMS (ESI-LTQ-Orbitrap): *m/z* calcd for $C_{345}H_{531}N_{73}O_{140}S_6$: $[M+6H]^{6+}$ 1355.5848, found: 1355.5871 (most intense isotope peak).

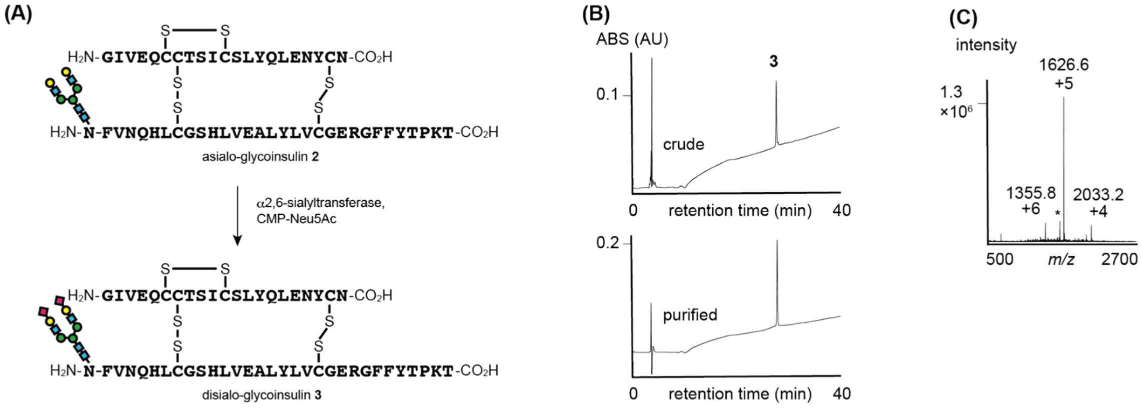

Figure S14. (A) Synthetic scheme of disialo-glycoinsulin **3**. (B) HPLC analysis of disialo-glycoinsulin **3**. (RT: 27.9 min, C4 column, 1% to 25% solvent B in solvent A over 10 min then 25% to 65% solvent B in solvent A over 30 min at 1 mL/min, absorbance at 218 nm) (C) Direct infusion ESI-MS analysis of disialo-glycoinsulin **3**. A peak with * corresponds to a fragmentation peak (without one sialic acid residue) during the MS analysis. Calcd Mass (average isotope composition): $[M+H]^{+}$: 8128.7, $[M+4H]^{4+}$: 2032.9, $[M+5H]^{5+}$: 1626.6, $[M+6H]^{6+}$: 1355.6.

2-15 Spectroscopic characterization (CD spectra)

Far-UV CD spectra were measured with a JASCO-J805 CD spectropolarimeter. Concentrations of proteins dissolved in distilled water were 7.4 μM for recombinant insulin, 8.4 μM for synthetic insulin **1**, 13.1 μM for asialo-glycoinsulin **2**, and 9.7 μM for disialo-glycoinsulin **3**.

2-16 Determination of disulfide linkages of synthetic insulin

To determine positions of disulfide bond formation, we performed enzymatic digestion of synthetic insulin using Glu-C (native endoproteinase from *Staphylococcus aureus*), which specifically hydrolyzes amide bonds at the carboxylic side of Glu in peptides, and compared the HPLC-retention times of the resulting peptide fragments with those of synthetic peptides (Fig. S14, 15, 16) because the native insulin has consecutive Cys residues (-Cys^{A6}-Cys^{A7}-).

To synthesize peptide standards which have different patterns of disulfide linkages, we first prepared short A-chain-I (short-A-I) **S11**, short A-chain-II (short-A-II) **S12**, short A-chain-III (short-A-III) **S13**, and short B-chain (short-B) **S14**. The peptides were synthesized by the flow platform at 60 °C using HBTU as a coupling reagent on the Fmoc-Glu(OtBu)-HMPA-ChemMatrix resin (50 μmol scale).

Short A-chain-I **S11**:
HRMS (ESI-TOF): *m/z* calcd for $C_{67}H_{111}N_{17}O_{24}S_3$: $[M+2H]^{2+}$ 816.8570, found: 816.8579.
Short A-chain-II **S12**:
HRMS (ESI-TOF): *m/z* calcd for $C_{67}H_{111}N_{17}O_{24}S_3$: $[M+2H]^{2+}$ 816.8570, found: 816.8630.
Short A-chain-III **S13**:
HRMS (ESI-TOF): *m/z* calcd for $C_{67}H_{111}N_{17}O_{24}S_3$: $[M+2H]^{2+}$ 816.8570, found: 816.8617.
Short B-chain **S14**:
HRMS (ESI-TOF): *m/z* calcd for $C_{70}H_{102}N_{21}O_{21}S_2$: $[M+H]^{+}$ 1636.6995, found: 1636.7028.

Representative protocol for disulfide bond formation of short A-chain and short B-chain

Short A-chain-I **S11** (1.8 mg, 1.1 μmol) and short B-chain **S14** (1.9 mg, 1.1 μmol) were dissolved in 0.1 M Tris-HCl buffer (pH 8.1, 1.13 mL) containing 6 M Gn-HCl. After 1 h, iodine (23 μmol) in AcOH (2.2 mL) was added and the mixture was agitated at room temperature. After 20 min, 1 M ascorbic acid (0.5 mL) was added, and the resulting mixture was diluted with distilled water (2 mL), filtered, and purified by RP-HPLC (Proteonavi, 4.6×250 mm, 0.1% aq TFA: 90% aq CH_3CN containing 0.1% TFA = 99:01 to 80:20 over 10 min then 80:20 to 40:60 over 30 min at 1 mL/min) to give peptide **S15** (white solid, 1.0 mg, 0.33 umol, 29%). HRMS (ESI-QTOF): *m/z* calcd for $C_{126}H_{196}N_{34}O_{41}S_4$: $[M+2H]^{2+}$ 1484.6585, found: 1484.6523.

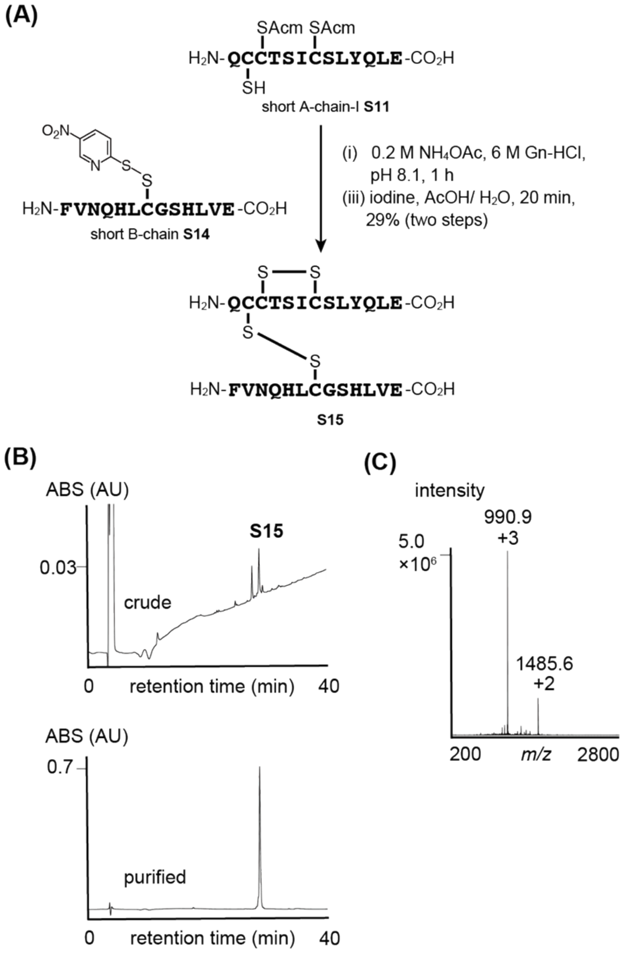

Figure S15. Disulfide bond formation of short A-chain-I **S11** and short B-chain **S14**. (A) Synthetic scheme. (B) HPLC analysis of peptide **S15**. (RT: 28.8 min, C4 column, 1% to 20% solvent B in solvent A over 10 min then 20% to 60% solvent B in solvent A over 30 min at 1 mL/min, absorbance at 218 nm) (C) Direct infusion ESI-MS analysis of peptide **S15**. Calcd Mass (average isotope composition): $[M+H]^+$: 2970.4, $[M+2H]^{2+}$: 1485.7, $[M+3H]^{3+}$: 990.8.

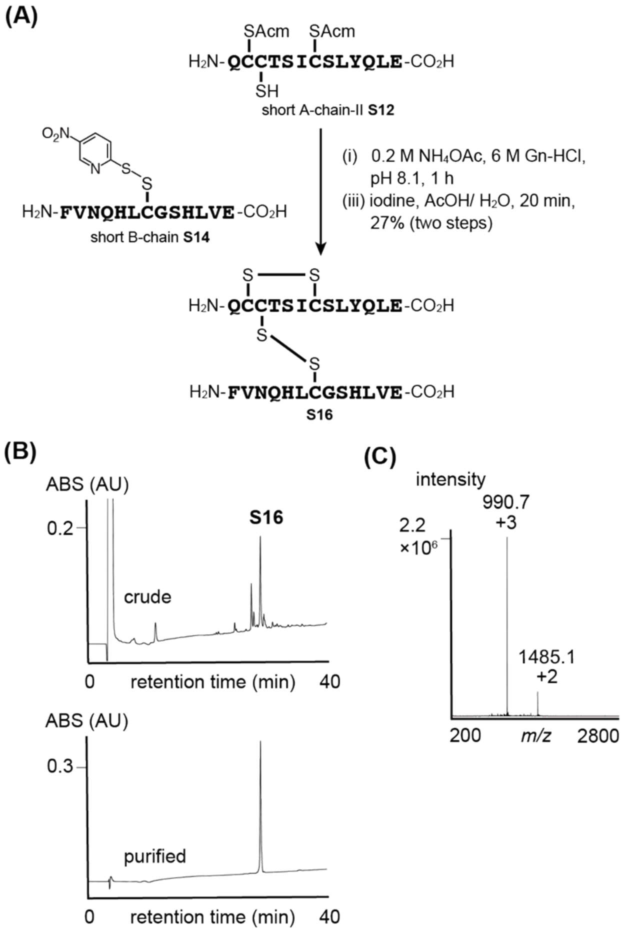

Figure S16. Disulfide bond formation of short A-chain-II **S12** and short B-chain **S14**. (A) Synthetic scheme. (B) HPLC analysis of peptide **S16**. (RT: 28.9 min, C4 column, 1% to 20% solvent B in solvent A over 10 min then 20% to 60% solvent B in solvent A over 30 min at 1 mL/min, absorbance at 218 nm) (C) Direct infusion ESI-MS analysis of peptide **S16**. HRMS (ESI-QTOF): *m/z* calcd for $C_{126}H_{196}N_{34}O_{41}S_4$: $[M+2H]^{2+}$ 1484.6585, found: 1484.6555. Calcd Mass (average isotope composition): $[M+H]^{+}$: 2970.4, $[M+2H]^{2+}$: 1485.7, $[M+3H]^{3+}$: 990.8.

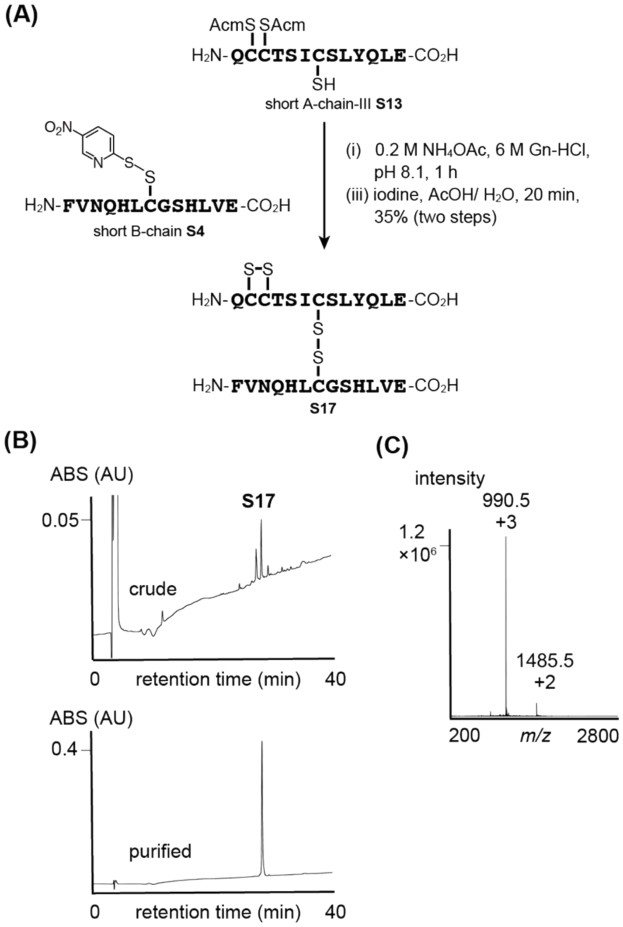

Figure S17. Disulfide bond formation of short A-chain-III **S13** and short B-chain **S14**. (A) Synthetic scheme. (B) HPLC analysis of peptide **S17**. (RT: 28.3 min, C4 column, 1% to 20% solvent B in solvent A over 10 min then 20% to 60% solvent B in solvent A over 30 min at 1 mL/min, absorbance at 218 nm) (C) Direct infusion ESI-MS analysis of peptide **S17**. HRMS (ESI-QTOF): *m/z* calcd for $C_{126}H_{196}N_{34}O_{41}S_4$: $[M+2H]^{2+}$ 1484.6585, found: 1484.6537. Calcd Mass (average isotope composition): $[M+H]^{+}$: 2970.4, $[M+2H]^{2+}$: 1485.7, $[M+3H]^{3+}$: 990.8.

Digestion of synthetic insulin 1

To the solution of synthetic insulin (10 μg, 1.7 nmol, final: 43 μM) in distilled water (20 μL), Glu-C (2.5 μg) in distilled water (5 μL), 1 M Tris-HCl (pH8.0, 1.6 μL, final: 40 mM) and distilled water (13.4 μL) was added. The mixture was incubated at room temperature for 9 h and then diluted with 50% aq CH_3CN containing 0.1% TFA. An aliquot of the resulting solution was used for RP-HPLC analysis (Proteonavi, 4.6×250 mm, 0.1% aq TFA: 90% aq CH_3CN containing 0.1% TFA = 99:01 to 80:20 over 10 min then 80:20 to 40:60 over 60 min at 1 mL/min).

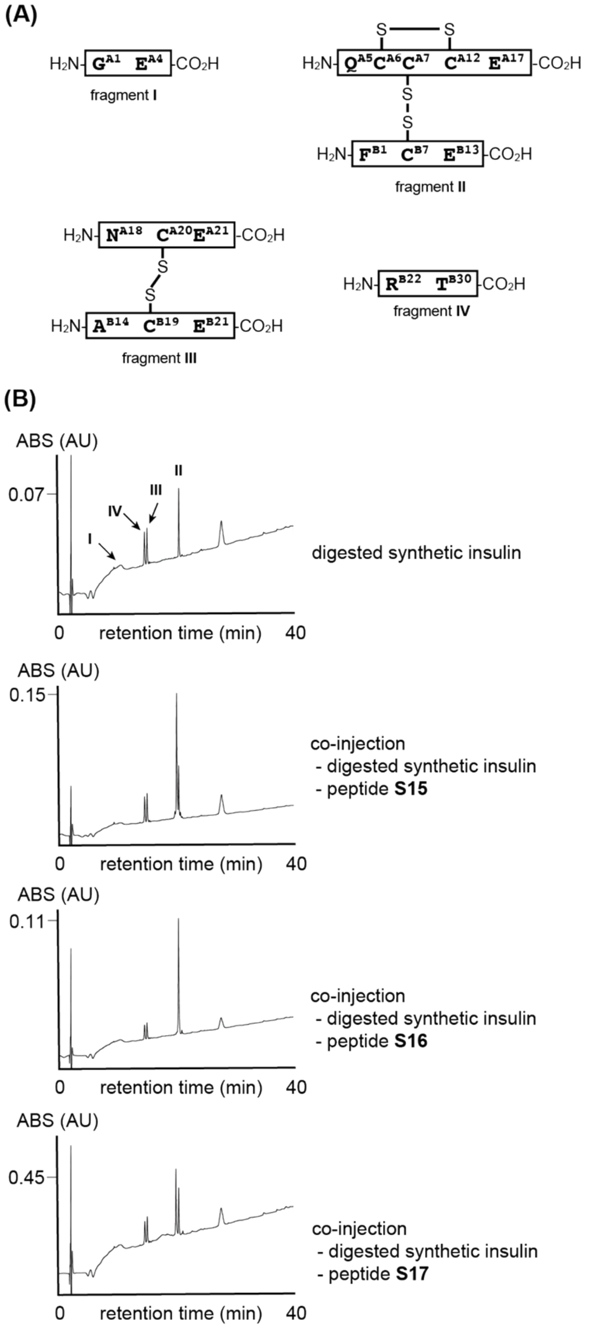

Figure S18. Glu-C digestion of synthetic insulin. (A) Structures of fragment peptides produced by peptidic digestion. (B) HPLC analysis of digested fragments (absorbance at 218 nm). The retention time of fragment **II** was confirmed to be identical with that of peptide standard **S16**. HRMS of fragment **I** (ESI-QTOF): *m/z* calcd for $C_{18}H_{33}N_4O_7$: $[M+H]^+$ 417.2344, found: 417.2341. HRMS of fragment **II** (ESI-QTOF): *m/z* calcd for $C_{126}H_{197}N_{34}O_{41}S_4$: $[M+3H]^{3+}$ 990.1081, found: 990.1048. HRMS of fragment **III** (ESI-QTOF): *m/z* calcd for $C_{59}H_{90}N_{14}O_{20}S_2$: $[M+2H]^{2+}$ 689.2943, found: 689.2924. HRMS of fragment **IV** (ESI-QTOF): *m/z* calcd for $C_{54}H_{79}N_{13}O_{13}$: $[M+2H]^{2+}$ 558.7954, found: 558.7936.

Digestion of asialo-glycoinsulin 2

Using the same protocol as above, the asialo-glycoinsulin was subjected to enzymatic digestion using Glu-C and analyzed by RP-HPLC (Proteonavi, 4.6×250 mm, 0.1% aq TFA: 90% aq CH_3CN containing 0.1% TFA = 99:01 to 80:20 over 10 min then 80:20 to 40:60 over 60 min at 1 mL/min).

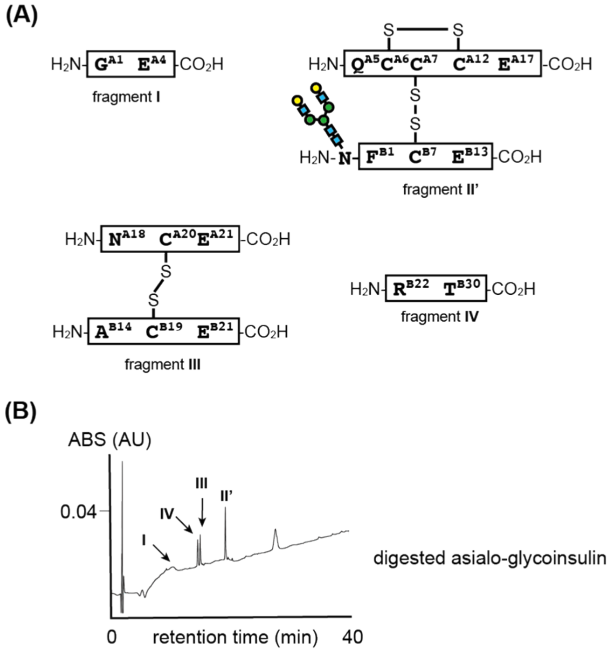

Figure S19. Glu-C digestion of asialo-glycoinsulin **2**. (A) Structures of fragment peptides produced by peptidic digestion. (B) HPLC analysis of digested fragments (absorbance at 218 nm). HRMS of fragment **I** (ESI-QTOF): *m/z* calcd for $C_{18}H_{33}N_4O_7$: $[M+H]^+$ 417.2344, found: 417.2343. HRMS of fragment **II'** (ESI-QTOF): *m/z* calcd for $C_{192}H_{306}N_{40}O_{88}S_4$: $[M+4H]^{4+}$ 1176.9890, found: 1176.9854. HRMS of fragment **III** (ESI-QTOF): *m/z* calcd for $C_{59}H_{90}N_{14}O_{20}S_2$: $[M+2H]^{2+}$ 689.2943, found: 689.2923. HRMS of fragment **IV** (ESI-QTOF): *m/z* calcd for $C_{54}H_{79}N_{13}O_{13}$: $[M+2H]^{2+}$ 558.7955, found: 558.7938.

2-17 Insulin receptor binding assay

Receptor binding was measured according to the previous reports.[ref.5] Briefly, IGF-IR-negative cells overexpressing the insulin receptor B (IR-B) or IR-A were serum-starved for 4 h before lysis. Lysates were captured in a 96 well plate, which was previously coated with anti-IR antibody 83-7. Approximately 500,000 fluorescent counts of europium-labelled insulin were added to each well along with varied concentrations of unlabeled competitor (insulin derivatives) and incubated for 16 h at 4 ^{o}C. After washing, time-resolved fluorescence was measured using 340 nm excitation and 612 nm emission filters with a BMG Lab technologies Polarstar fluorometer (Mornington,

Australia). Insulin and synthetic analogues curves are from four separate experiments with each point performed in triplicate.

3. References

(1) M. D. Simon, P. L. Heider, A. Adamo, A. A. Vinogradov, S. K. Mong, X. Li, T. Berger, R. L. Policarpo, C. Zhang, Y. Zou, X. Liao, A. M. Spokoyny, K. F. Jensen, B. L. Pentelute, *ChemBioChem*, 2014, **15**, 713.

(2) Y. Kajihara, Y. Suzuki, N. Yamamoto, K. Sasaki, T. Sakakibara, L. R. Juneja, *Chem. – A. Eur. J.*, 2004, **10**, 971.

(3) M. Murakami, R. Okamoto, M. Izumi, Y. Kajihara, *Angew. Chem. Int. Ed.* 2012, **51**, 3567.

(4) T. Yamamoto, M. Nakashizuka, H. Kodama, Y. Kajihara, I. Terada, *J. Biochem.* 1996, **120**, 104.

(5) M. A. Hossain, R. Okamoto, J. A. Karas, P. Praveen, M. Liu, B. E. Forbes, J. D. Wade, Y. Kajihara, *J. Am. Chem. Soc.*, 2020, **142**, 1164.
